# Pinpointing Cu-Coordination Motifs in Bio-Inspired MOFs by Combining DFT-Assisted XAS Analysis and Multivariate Curve Resolution

**DOI:** 10.1021/acs.jpcc.4c08029

**Published:** 2025-02-06

**Authors:** Beatrice Garetto, Ning Cao, Valeria Finelli, Erlend Aunan, Matteo Signorile, Unni Olsbye, Silvia Bordiga, Ainara Nova, Elisa Borfecchia

**Affiliations:** † Department of Chemistry, NIS and INSTM Reference Centre, Università di Torino, Via G. Quarello 15/A, I-10135, and Via P. Giuria 7, I-10125 Turin, Italy; ‡ SMN Centre for Material Science and Nanotechnology, Department of Chemistry, University of Oslo, N-0315 Oslo, Norway; § Hylleraas Centre for Quantum Molecular Sciences, Department of Chemistry, University of Oslo, P.O. Box 1033, Blindern, N-0315 Oslo, Norway; ∥ University School of Advanced Studies, IUSS Pavia, Palazzo del Broletto, P.zza della Vittoria 15, I-27100 Pavia, Italy

## Abstract

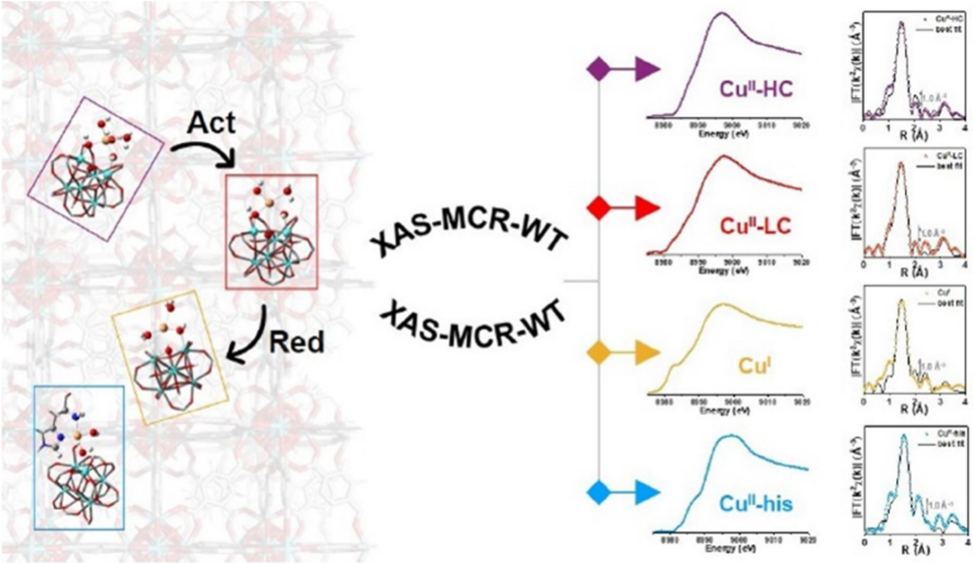

In recent years, X-ray absorption spectroscopy (XAS) has emerged as an essential technique for investigating the structure and composition of heterogeneous catalysts, providing valuable insights into the nature of active sites within these systems. However, the average nature of the XAS signal, inherently merged over all the absorber-containing species forming during in situ/operando experiments, often complicates the interpretation of the data. Nonetheless, advanced analysis methods have been developed to address this problem. In particular, herein we employed in situ XAS spectroscopy together with multivariate curve resolution-alternating least squares (MCR-ALS) and wavelet transform (WT) analysis to monitor the evolution of distinct Cu species in histidine-modified Cu-UiO-66 MOFs and to obtain detailed structural insights about the Cu sites. A novel approach adopted in this work was the application of density functional theory (DFT)-assisted extended X-ray absorption fine structure (EXAFS) fitting to quantitatively refine the local structure of the MCR-derived pure Cu species. This approach revealed the preferential redox activity of Cu^II^ ions coordinated within the defective Zr clusters of the MOF, compared to Cu^II^ ions bound to both the histidine molecule and the defective site during a standard redox reaction protocol.

## 1 Introduction

X-ray absorption spectroscopy (XAS) is a highly sensitive and widely utilized technique for characterizing the electronic and structural properties of materials.^1−3^ By probing the local atomic environment of specific elements, XAS offers detailed insights into oxidation states, coordination geometries, and interatomic distances.^4−6^ This level of accuracy makes it indispensable across diverse fields, including materials science, chemistry, and environmental studies.^7−10^ Its capacity to deliver element-specific information at the atomic scale is particularly advantageous for analyzing complex systems like zeolites and metal–organic frameworks (MOFs), where understanding the atomic structure of active sites is critical. These systems are often involved in key catalytic processes, such as methane-to-methanol oxidation,^11^ the water-gas shift reaction,^12^ and selective catalytic reduction (SCR) of NO_*x*_,^13^ where the detailed knowledge of local atomic environments is essential for optimizing performance. XAS is element-selective, but the signal reflects an average response from all absorber-containing species within the sample volume probed by the X-ray beam, weighted by their relative abundance.^14−16^ This averaging effect presents significant challenges when multiple absorber-containing species coexist and evolve dynamically throughout the experiment. Such scenarios are common during in situ and operando studies of catalysts, which have become the golden standard for characterizing catalytic processes.

To address these limitations, the use of machine learning-based methods like the unsupervised multivariate curve resolution-alternating least squares (MCR-ALS) has emerged as a powerful tool to enhance the analysis of XAS data.^17,18^ According to the literature,^7,17,19^ this approach enables the decomposition of the original spectral data set into chemically and physically meaningful “pure” spectra (where the term “pure” is employed within the framework of the methodological limitations), along with their corresponding concentration profiles. MCR-ALS provides critical insights into the speciation of active sites in catalytic systems by isolating individual spectral components, overcoming the averaging limitations of conventional XAS analysis. First introduced by Tauler, this methodology has been widely applied across various fields over the last two decades.^20−22^ While MCR-ALS is commonly used for X-ray absorption near edge structure (XANES) analysis, as evidenced by Skorynina et al.,^23^ its application in the extended X-ray absorption fine structure (EXAFS) spectral region is less frequent. Extracting EXAFS spectra for pure species using this method remains rare due to the complexity and typically higher noise, as well as to the strong dependency of the EXAFS equation on the temperature.^24,25^ Nonetheless, when successful, it offers a deeper understanding of local atomic environments and structural dynamics that would otherwise be difficult to obtain. As demonstrated in a recent study, Kvande et al. were able to decompose both the XANES and EXAFS regions of the XAS spectra for pure Cu species present in Cu-MOR zeolites during a temperature-programmed reduction (TPR) experiment.^7^ This achievement provided valuable insight into the speciation of copper in the system. However, due to the high temperatures employed in their experiments, the quality of the MCR-derived EXAFS spectra was insufficient to perform a detailed structural refinement analysis. Clearly, the experimental temperature plays a crucial role in determining the EXAFS quality of the spectra.

In this work, we investigated, primarily through the MCR analysis, the speciation and evolution of Cu sites during a well-designed redox experiment in histidine-modified Cu-UiO-66 MOFs, which were synthesized, initially characterized in their as-prepared form and tested toward the partial oxidation of cyclohexene in the liquid phase by Aunan et al.^26^ These bioinspired catalysts are designed to mimic the active sites of cuproenzymes, particularly lytic polysaccharide monooxygenases (LPMOs), which are known for their ability to catalyze the partial oxidation of strong C–H bonds under ambient conditions.^27,28^ The active motif in these enzymes features a mononuclear copper ion coordinated by two histidine ligands, a structural element that has garnered significant attention from the scientific community due to its potential in sustainable catalysis.^29^ The role of the histidine ligand is to provide stabilization of both Cu^I^ and Cu^II^ states while still maintaining open coordination sites on Cu to allow for O_2_ or H_2_O_2_ to interact in a controlled manner.^30−32^ MOFs provide a promising platform for incorporating copper and histidine, with Zr-based UiO-66 being a particularly attractive candidate due to its exceptional physicochemical stability and versatility.^33−36^

In this study, we monitored two representative Cu-UiO-66 MOF samples under model conditions to evaluate the redox properties of these catalysts, with the aim of gaining insights relevant to their potential application in light alkane oxidation reactions in the gas phase. The relatively low temperature (150 °C) maintained during the experiment allowed us to obtain high-quality MCR-derived pure Cu spectra using the so-called *Waterfall approach* to analyze both the XANES and EXAFS regions of the spectra. Additionally, we succeeded in pinpointing the local structural properties of the different Cu sites/species present in the materials by using DFT-assisted EXAFS fitting analysis and TD-DFT calculations,^37^ complemented by advanced data analysis techniques such as wavelet transform (WT) analysis.^38,39^ WT was particularly useful for detecting multimeric species and discriminating between different scattering contributions in the high-R region of the EXAFS spectra, providing a more detailed understanding of the Cu coordination environment.^40^ Furthermore, we evaluated the energy of the reaction intermediates that could be involved in the Cu^II^ reduction by H_2_ obtained using DFT methods. These results provided insight into the reduction properties of different Cu species located on Zr defective sites.

## 2 Materials and Methods

### 2.1 Histidine-Modified Cu-UiO-66 MOFs

The materials reported in this paper have been synthesized and preliminary characterized in previous studies. Details about the synthetic procedure and composition can be found in the work conducted by Aunan et al. The main compositional and physicochemical properties of the investigated sample are also reported in Supporting Information, Table S1, adapted from Aunan et al.^26^ In summary, two materials will be studied, referred to hereafter as His-Cu-1 and His-Cu-2, characterized by Cu:His ratios of 2.6 and 7, respectively.

### 2.2 Experimental Setup and Collection Methods for XAS Analysis

The two examined samples underwent characterization through in situ XAS experiments at the Cu K-edge, conducted at the BM23 beamline of the European Synchrotron Radiation Facilities (ESRF).^41,42^ The powdered His-Cu-1 and His-Cu-2 samples were initially prepared in the form of self-supporting pellets of 1.3 cm diameter and subsequently positioned within the Microtomo reactor cell, a specialized cell for in situ/operando experiments under controlled conditions.^43^ The cell is equipped with a custom gas flow system, comprising three primary channels, each linked to gas bottles with distinct compositions, specifically pure oxygen (O_2_), pure helium (He), and pure hydrogen (H_2_). The presence of three mass flow controllers is essential for regulating the overall flow rate. In addition, the cell is connected to a vacuum line integrated into the beamline, facilitating a rapid transition between dynamic and static conditions and mitigating the risk of dangerous gas mixtures. During the experiment, the effluent was analyzed with an online mass spectrometer, and the sample temperature was controlled and monitored by the integrated heating system within the Microtomo cell.

**Figure 1:**
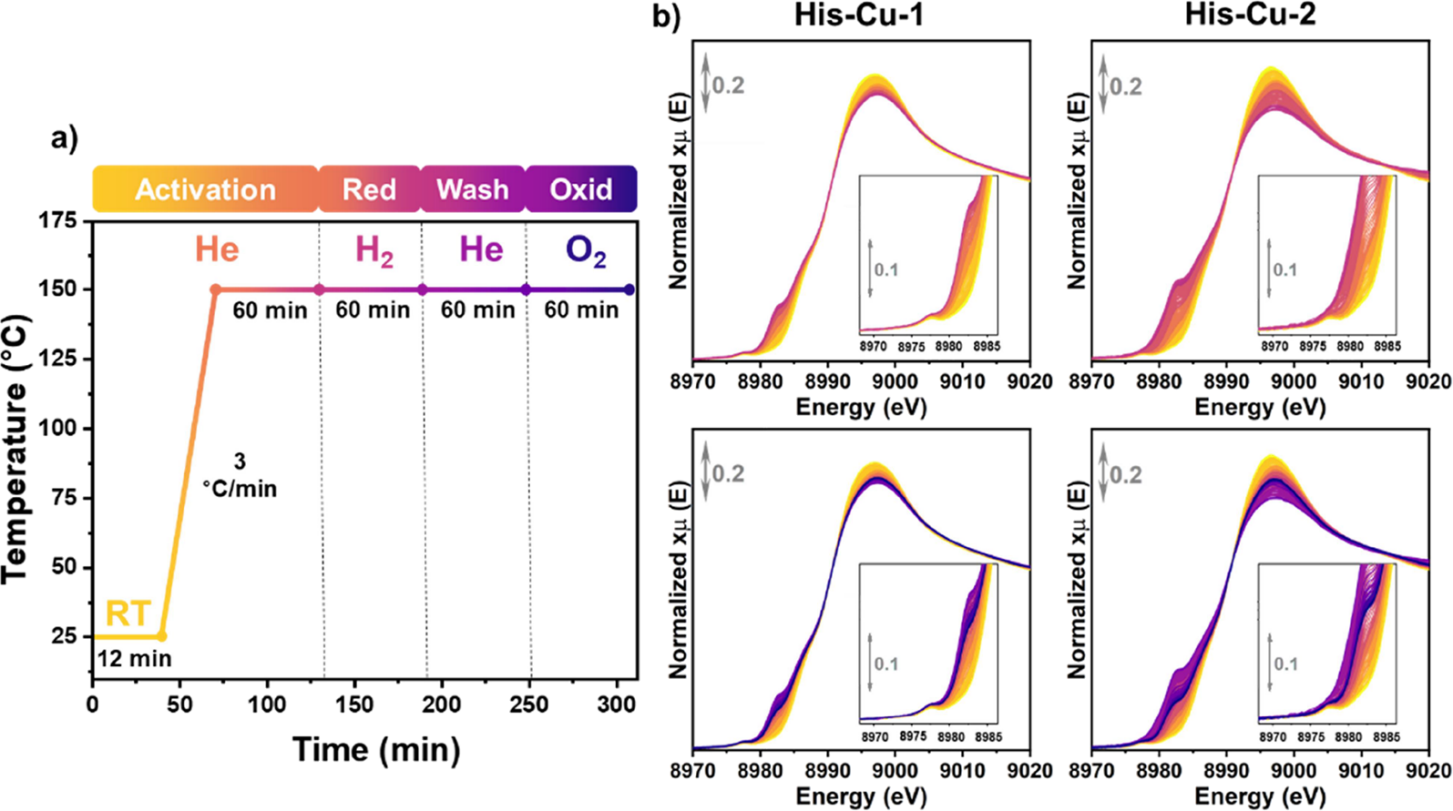
(a) Experimental protocol for in situ XAS over His-Cu samples. It consists of activation in helium at 150 °C with a temperature ramp of ca. 3 °C/min (**Activation**), reduction (**Red**) in hydrogen, a helium wash step (**Wash**), and oxidation in oxygen (**Oxid**). The acquisition rate is 2.5 scan/min during the whole experiment (b) XANES spectra for each examined sample. On the top panel are reported the spectra collected in the activation and reduction steps, while in the bottom panel, the oxidation step is also involved. In the inset is reported a magnification of the 1s → 3d transition signals related to Cu^II^. The adopted color code for the different treatment steps is the same as indicated in the protocol scheme in part (a).

Aiming to evaluate the redox activity of the samples, an in situ standard reaction protocol was applied (Figure 1a). Initially, the samples were thermally treated under inert conditions, heated at 3 °C/min from room temperature (RT) up to 150 °C, and kept in these conditions for 1 h. This preliminary step is crucial as it enables the removal of water molecules adsorbed within the MOF pores. Afterward, we shifted the gas feed from an inert to a reducing environment, initiating the flow of H_2_ and maintaining it for 1 h. During this step, the Cu^II^ sites initially present in the as-prepared materials are expected to undergo reduction to Cu^I^,^26^ resulting in a change in their oxidation state and coordination geometry. After the reduction step in H_2_, He gas is reintroduced to eliminate potential contaminants (this step will be called “He-wash” later), followed by the last step in O_2_ for 1 h. Investigating the reoxidation process sheds light on the reversibility and stability of the Cu sites, relevant factors for assessing the long-term performance and durability of the material in various applications.

In situ Cu K-edge XAS measurements were conducted in transmission mode, employing a water-cooled double-crystal Si(111) monochromator for scanning the incident energy. Detection of incident (I_0_) and transmitted photons (I_1_) is carried out using ionization chambers filled with a He/Ar mixture.^44^ All the experimental XAS spectra were acquired with an acquisition time of approximately 2.5 min/scan. Subsequently, normalization to the unity edge jump at the Cu K-edge was performed using the Athena software from the Demeter suite.^45^ Fourier-transformed (FT-) EXAFS spectra were obtained from the k^2^-weighted data in the 2.5–11.0 Å^–1^ k-range.

### 2.3 XAS Data Analysis Methods

#### 2.3.1 MCR-ALS Analysis

In the realm of XAS, the use of MCR-ALS analysis is progressively expanding, owing to its capability to isolate pure components from complex mixtures of chemical species. The method is grounded on resolving a mixture of spectra, represented by a matrix D, into its pure contributions, such as spectra (S) and concentration (C) matrices: D = CxS^T^.^46−48^ These can be derived employing various methods, such as singular value decomposition (SVD), non-negative matrix factorization (NMF), or principal component analysis (PCA). Initially, the number of principal components (PCs) must be determined through critical evaluation of the result in terms of spectroscopic and chemical-physical meaningfulness, or a priori with statistical estimators.^17^ Afterward, the algorithm developed by Tauler and co-workers^22^ alternates between two main steps: alternating least-squares (ALS) and multivariate curve resolution (MCR). In the ALS step, the pure spectra and the concentration profiles are alternately optimized using the least-squares method, followed by a new optimization of the spectra until either convergence or the set iteration limit is reached. This iterative process persists until convergence criteria are satisfied. Importantly, to assess the accuracy of the reconstructed spectra relative to the measured data, various metrics are used, such as the Lack of Fit (LoF) and the explained variance.^17^ Ultimately, the generation of pure components to be used as initial guesses in the iterative reconstruction is efficiently managed by specific methods, such as SIMPLISMA.^49^

In this work, for both the samples, the XANES and the EXAFS spectral regions are considered in the analysis, involving the whole experimental energy range (8800–9727 eV). Aiming to more sensitive and accurate results, a step-by-step procedure is followed, called hereafter as the *Waterfall approach*. In this context, the normalized μ(E) XAS spectra related to a sub-step of the experimental protocol are considered, from which a number N of pure species is extracted. Afterward, the obtained spectra are inserted into a larger data set as references to guide the iteration. In this way, a number *N* + 1 of pure species can be obtained. This procedure continues until the whole data collection is inserted into a unique MCR analysis. Importantly, the *Waterfall approach* has specific requirements: (a) an appropriate statistical variance, dictated by the number of experimental spectra considered; (b) physical and chemical sense of the results. For this analysis, we used the MATLAB-based MCR-ALS graphical user interface (GUI) developed by Jaumot et al., employing Matlab R2022a.^50^ For further details on the ALS quality control parameters, the interested reader is referred to Supporting Information, Table S2.

#### 2.3.2 WT EXAFS Analysis

FT-EXAFS signals represent single or multiple scattering contributions between the absorber and the neighboring atoms.^38^ Consequently, information regarding the local structure and coordination environment around the investigated metal center can be obtained. However, when the scattering contributions originate from neighboring atoms localized within similar coordination spheres, especially in the 2.5–3.5 Å range, their signal can overlap in the FT-EXAFS space, becoming indistinguishable.^39,51^ The WT approach can be employed to offer a more robust description of high-R EXAFS features. Its sensitivity to the chemical nature of the scatterers surrounding the absorber is enhanced by exploiting the scatterer Z-dependency in the backscattering amplitude functions, *F*(*k*).^52^ The typical result of the analysis is a 2D representation, showing simultaneous k- and R-space spectral features for better signal discrimination.^37,40^

The WTs maps showed in this work are obtained from the so-called Morlet function:
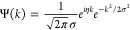


More precisely, the wavelet resolution is regulated by the η value, which represents the frequency of a sine wave convoluted by a Gaussian curve with σ as the standard deviation. Therefore, regulating these parameters allows the operator to achieve optimal visualization of the desired spectral features, even though equally high resolution in both k- and R-space is not achievable. According to recent literature, the best resolution condition at a given distance of interest is obtained by choosing σ = 1 and η = 7 for all the WT representations.

The selection of the appropriate mother function, which strongly depends on the type of analysis, is crucial. Additionally, the effect of constructive and destructive interference of the backscattered waves can result in the appearance of multiple lobes in the WT maps, which can correspond to a single group of atoms with identical atomic numbers.^53^

#### 2.3.3 DFT-Assisted EXAFS Fitting Analysis on Pure EXAFS Spectra

A critical step to deepen the structural information on Cu centers embedded within the investigated MOFs involves employing EXAFS fitting analysis. This method is instrumental in deciphering the local structural properties of the absorber atom by fitting the experimental EXAFS data into theoretical models.^54^ It allows the computation of photoelectron scattering parameters for individual scattering paths. Both single and multiple scattering paths (SS and MS, respectively) are thus parametrized using structural parameters, with values determined to minimize discrepancies between the model predictions and experimental spectra.^37^

Optimized geometries with periodic DFT methods (see Section 3.3) were used for the analysis to describe more accurately the MCR-derived pure Cu spectra obtained through the *Waterfall approach* previously explained. The analysis was conducted using Artemis software, a GUI from the Demeter package implementing the FEFF6 code for ab initio calculation.^45^ For the Fourier transform (FT), the k^2^-weighting was applied within the k range of 2.5–11 Å^–1^, with fitting the 1 5 Å R-space region.

### 2.4 Computational Methods

Geometry optimizations using cluster models were conducted with the Gaussian 16 package.^55^ Density functional theory (DFT) was employed with the PBE0 functional^56^ including the D3 version of Grimme’s empirical dispersion correction with Becke–Johnson damping,^57^ and a def2-SVP basis set.^58^ All energy values calculated at PBE0-D3(BJ)/def2-SVP were refined using a larger def2-TZVP basis set.^59^ To keep the rigidity of the MOFs, the carbon atoms in the carboxylate ends were frozen during geometry optimizations.

For periodic models, all calculations were conducted using the CP2K package^60^ with the PBE functional and a mixed DZVP Gaussian and auxiliary plane-wave basis set.^61^ Grimme’s D3 dispersion model was employed to account for dispersion forces.^57^ The unit cell of UiO-66 was taken from the Cambridge Crystallographic Data Centre,^62^ with the following unit cell dimension: *a* = *b* = *c* = 20.913 Å, α = β = γ = 90°.

All time-dependent density functional theory (TDDFT) calculations were performed using the ORCA electronic structure package 5.0.^63^ The CAM-B3LYP range-separated functional was selected for its suitability in excited state calculations.^64^ The zeroth-order regular approximation (ZORA) was employed to account for scalar relativistic effects.^65^ The scalar relativistic basis set ZORA-def2-TZVP was used for all atoms except Cu and Zr, where SARC-ZORA-TZVP and ZORA-def2-TZVPP basis sets were applied, respectively.^66^ The calculations utilized the resolution of identity (RI-J) algorithm for the Coulomb term^67^ and the ‘chain of spheres exchange’ (COSX) algorithm for the exchange term,^68^ with a tight self-consistent field (SCF) convergence threshold. To align with experimental XAS data, several reference Cu complexes were selected^63−65^ and their XANES spectra were simulated using our protocol (results in Supporting Information, Table S3). By comparing the pre-edge positions between experimental and simulated XAS, a calibration shift of −2.9 eV was determined. Consequently, all TDDFT-calculated XAS spectra for our system were shifted by −2.9 eV to ensure alignment in the plotted results.

## 3 Results and Discussion

### 3.1 From Qualitative to Quantitative XAS Analysis

#### 3.1.1 In Situ XAS Spectra

Figure 1b presents the experimental Cu K-edge XAS spectra of His-Cu-1 and His-Cu-2, collected throughout the entire experiment in accordance with the protocol outlined in Figure 1a. Previous studies by Aunan et al.^26^ have identified distinct Cu centers in these materials, primarily consisting of Cu^II^ complexes either coordinated to defective nodes or linked to histidine residues. As expected, in Figure 1 (top), it is visible that both samples exhibit the dipole-forbidden 1s → 3d pre-edge peak, characteristic of the Cu^II^ oxidation state at RT under an inert atmosphere. Minor variations in the white line (WL) shape, reasonably attributable to differences in the Cu coordination environment, are observed comparing the two samples. As the temperature increases from RT to 150 °C, a gradual decrease in the WL intensity is observed, which can be correlated with a reduction in the Cu coordination number due to the desorption of physisorbed water molecules. Upon switching the gas feed from He to H_2_, the reduction of Cu is visible in both samples. Spectroscopically, this is indicated by the progressive decrease of the 8977 eV pre-edge peak, paralleled by the development of a prominent dipole-allowed 1s → 4p edge peak located at ca. 8983 eV, along with a further reduction in WL intensity.

Interestingly, when the environment is switched from H_2_ to O_2_ gas (see Figure 1, bottom), partial reoxidation of the Cu sites is observed in both samples, as evidenced by the intensity decrease of the Cu^I^ 1s → 4p peak. Nonetheless, after the experiment, such a peak is still clearly visible, suggesting the presence of a mixed Cu^II^/Cu^I^ state under the adopted experimental conditions. This behavior can be attributed to the limited oxidizing capability of molecular oxygen and the insufficient exposure time for the complete oxidation of Cu^I^ to Cu^II^.

Based on these observations, we can safely state that both samples exhibited redox activity throughout the experiment, albeit with some differences. A further comparison of the two samples reveals that, at the end of the reduction step, the intensity of the 1s → 4p transition peak associated with Cu^I^ centers is higher in the His-Cu-2 sample. This suggests the formation of Cu^I^ sites to a greater extent during the reduction process, further corroborated by the complete disappearance of the 1s → 3d peak in this sample, indicative of the reduction of all Cu^II^ species. Additionally, significant changes in the WL intensity in the His-Cu-2 sample suggest substantial geometrical rearrangements at the Cu sites and a reduction in coordination number, characteristic of Cu^I^ species, further supporting this hypothesis.

#### 3.1.2 MCR Results

**Figure 2:**
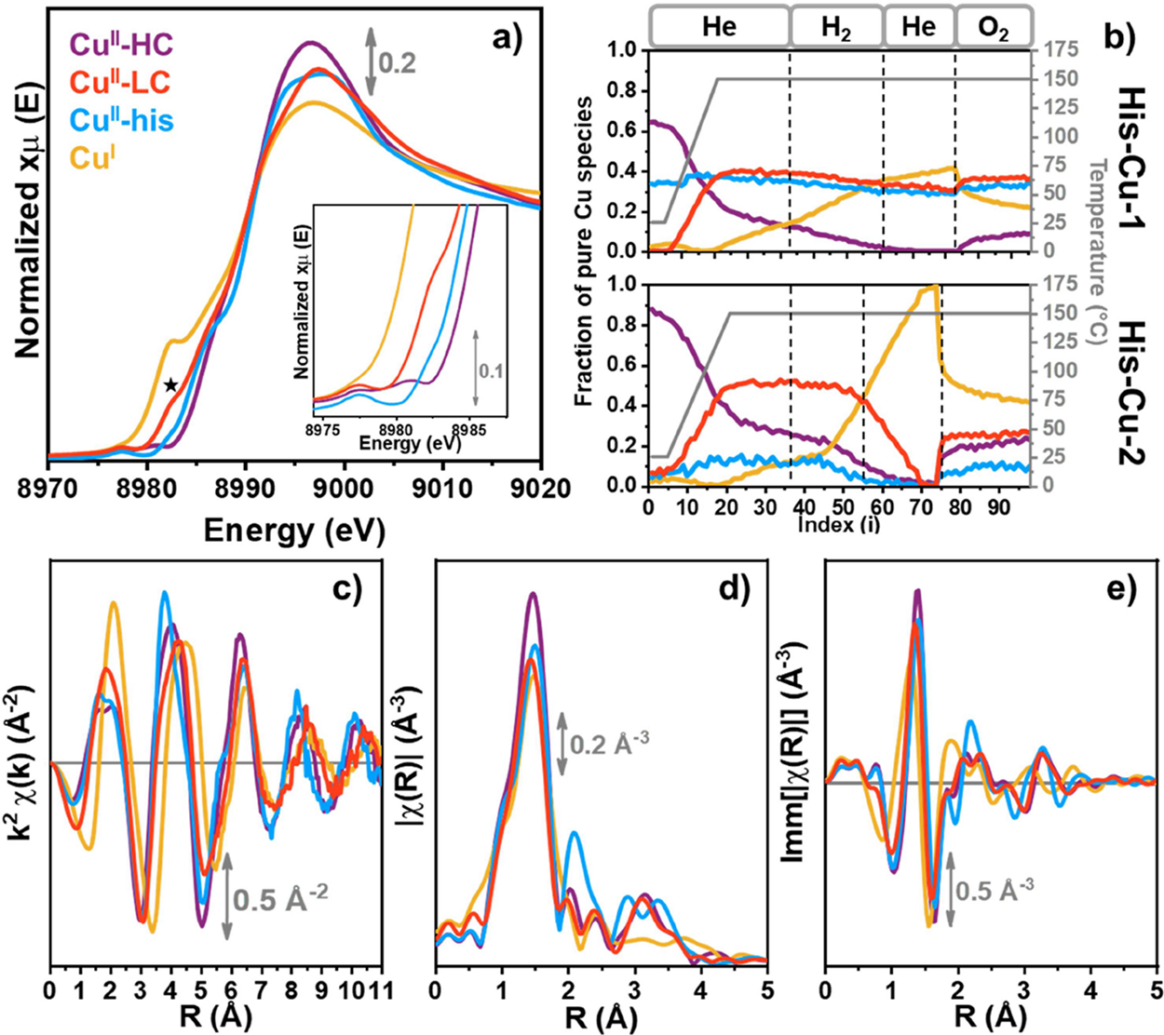
(a) XANES spectra of the pure Cu species obtained by MCR analysis, carried out following the *Waterfall approach* explained in the Methods section. The * symbol indicates an MCR reconstruction artifact. In the inset is reported a magnification of the 1s → 3d transition signals related to Cu^II^. (b) Corresponding temperature-dependent concentration profiles for each Cu species found in both the systems. (c) MCR-derived k-space EXAFS spectra for the pure Cu species identified. The k range used to calculate the FT is 2.5–11 Å^–1^. (d,e) Corresponding magnitude (d) and imaginary (e) parts of the phase-uncorrected FT-EXAFS spectra in R-space, obtained by Fourier transforming the *k*^2^χ(*k*) curves reported in (c).

The estimation of the number of distinguishable chemical species contributing to the XANES spectral variations observed in Figure 1, as well as the subsequent isolation of their respective spectral contributions, was achieved through MCR-ALS analysis. Following the *Waterfall approach* detailed in the Methods section, four distinct pure Cu species were finally identified in the global data set entailing XAS spectra for both samples collected through the whole protocol illustrated in Figure 1a. The corresponding pure XANES spectra are presented in Figure 2a, while their concentration profiles are depicted in Figure 2b.

First, focusing on the RT static state in He, two distinct Cu^II^ species (purple and light blue spectra) are copresent in the mixture, exhibiting substantial differences in their XANES spectral features. In particular, their intensity and shape of the WL peak, together with the presence of a more pronounced Cu^II^ 1s → 4p transition peak in the blue species, suggest the existence of structurally and chemically distinct Cu^II^ species. Moreover, the concentration profiles reveal different fractions of these species within the samples, which are notably consistent with the postsynthesis compositional data for Cu and histidine fractions, as reported in the Supporting Information, Table S1, as well as with previously reported EXAFS analysis of the as-prepared materials.^26^

Based on these observations and supported by the previous study conducted by Aunan et al., we designate these two species as **Cu**^**II**^**–HC** (Higher Coordination), referring to Cu^II^ species coordinated to the defective Zr-cluster of the MOF, and **Cu**^**II**^**-his**, representing Cu^II^ centers coordinated by both the defective Zr-cluster and a histidine unit. During the activation step in He, two new species (red and yellow spectra) emerge at the expense of **Cu**^**II**^**-HC**, while the **Cu**^**II**^**-his** component remains relatively unvaried in both samples. The red XANES spectrum shares spectral similarities with the **Cu**^**II**^**–HC**, except for a reduced WL peak intensity. The newly formed species is termed **Cu**^**II**^**-LC** (Lower Coordination), representing a Cu site with a reduced coordination number, likely due to the desorption of an additional water molecule from the **Cu**^**II**^**–HC** center. Conversely, the yellow species, which forms in minor quantities, is defined as a **Cu**^**I**^ site, since no detectable traces of Cu^II^ 1s → 3d electronic transition in the XANES spectral region are present. This earlier reduction behavior can be attributed to the well-documented “self-reduction” effect of the Cu sites, a phenomenon particularly observed in other porous systems, such as zeolites.^69^

Considering the reduction step of the experiment, the formation dynamics and relative abundance of the **Cu**^**I**^ sites differ between the samples. Consistently with observations from the step in inert gas flow at 150 °C, the formation of **Cu**^**I**^ species is more pronounced in the His-Cu-2 sample. After the reduction step, O_2_ is flushed in the system, resulting in only a partial restoration of the initial Cu^II^ species, as the Cu^I^ fraction does not return to zero by the end of the experiment.

These conclusions are fully consistent with the qualitative observations made in the previous section. Moreover, it is evident that, throughout the experiment, the **Cu**^**II**^**-his** component remains largely inert, exhibiting only minor variations within the 5–10% total Cu error margin of the MCR method. In contrast, the cluster-derived species are actively involved in the redox process, leading to the partial conclusion that the **Cu**^**I**^ species predominantly form from the **Cu**^**II**^**-HC** and **Cu**^**II**^**-LC** sites. Additionally, given that the formed fraction of **Cu**^**I**^ species is higher in the sample with a lower histidine content, it is likely that the presence of this anchoring site influences the redox properties of the material.

By analyzing the MCR-derived EXAFS region during the experiment, additional insights into the local environment of Cu ions were obtained. As the MCR reconstruction was applied to the entire energy range, the EXAFS spectra were directly extracted from the MCR-derived μ(E) spectra and visualized in k-space (Figure 2c) and in R-space, considering both magnitude (Figure 2d) and imaginary parts (Figure 2e) of the FT.^70^ In k-space, while the oscillations are similar in all the cases, minor differences are observed at higher k-values, with decreased intensities, in particular for **Cu**^**II**^**-LC** and **Cu**^**I**^. This can be attributed to the Debye–Waller factor contained in the EXAFS equation, which is known to dampen the oscillation intensity as the temperature increases, with these species forming at 150 °C.^25^ Moreover, in line with the XANES features discussed before, a lower coordination number in the first coordination sphere is also expected for these two Cu-species, also resulting in a lower amplitude of the EXAFS oscillations.

The most prominent feature in the FT-EXAFS spectra, visible in Figure 2d, is the so-called first-shell peak, which corresponds to the single-scattering (SS) contributions involving extra-ligand light atoms, such as oxygen from water adsorbed molecules and framework atoms from the MOF scaffold. The spectra indicate that **Cu**^**II**^**-HC** and **Cu**^**II**^**-his** exhibit higher peak intensities, likely reflecting higher Cu coordination numbers, consistent with the proposed speciation. Interestingly, well-defined second and third shell peaks are observable for the Cu^II^ pure species. While the spectra of **Cu**^**II**^**–HC** and **Cu**^**II**^**-LC** largely overlap, suggesting a similar Cu coordination environment, the **Cu**^**II**^**-his** species displays distinct peaks, which could be consistent with the histidine unit coordinating the Cu centers. In the **Cu**^**I**^ species, the third-shell region is completely dampened, suggesting a higher degree of structural disorder in the local environment of the Cu^I^ cation. This structural disorder likely leads to destructive interference between the EXAFS scattering paths, similar to what was previously observed for Cu^I^ sites in zeolites.^19,71^

In Figure 2e, the imaginary part of the R-space is particularly informative for detecting the presence of higher atomic number (*Z*) elements. Notably, at approximately 3.25 Å, a well-defined oscillation is observed for all the Cu^II^ species, which can be attributed to the presence of Zr atoms within the MOF cluster. To further confirm these assumptions, WT analysis was performed on the MCR-derived EXAFS spectra.

#### 3.1.3 WT Results

**Figure 3:**
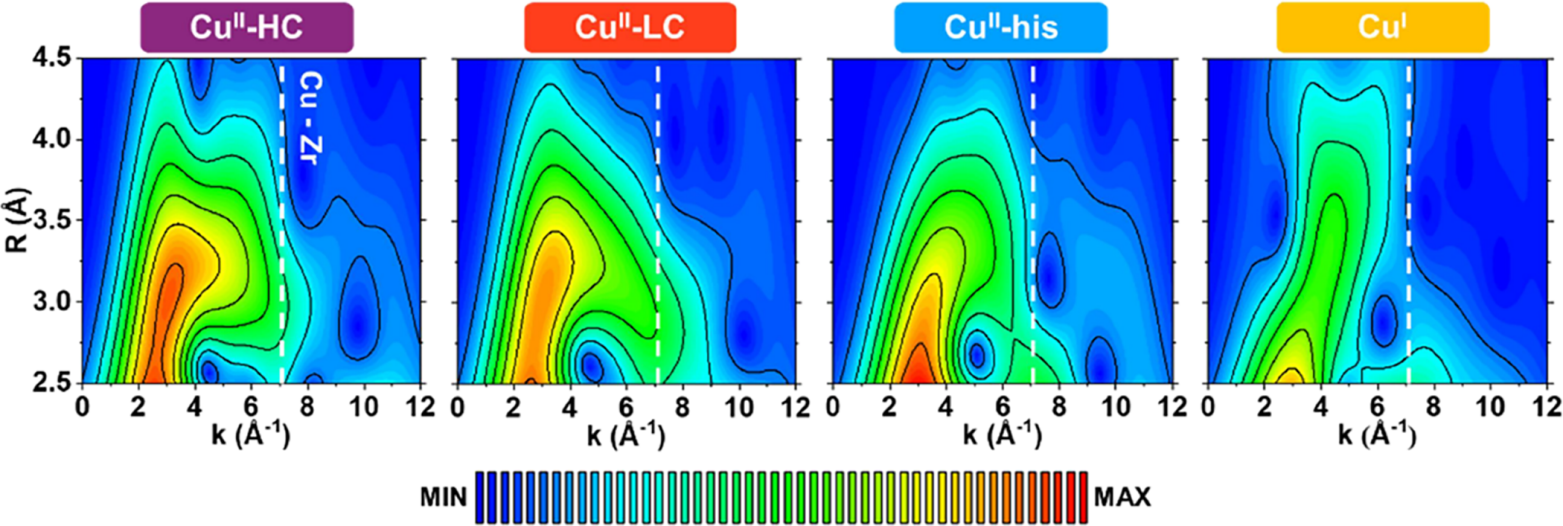
WT maps obtained by WT-EXAFS analysis for pure Cu^II^ and Cu^I^ species resolved by MCR analysis. In the first WT map, the Cu–Zr contribution is reported at 7 Å^–1^, meanwhile in the others, it is indicated with the white dashed line. The color scale associated with the WT intensity is reported at the bottom of the figure.

Given its sensitivity to the chemical nature of the scatterers surrounding the absorbing atom, WT is a valuable tool for confirming our hypothesis regarding the structural morphology of the MCR-derived pure Cu species. Accordingly, WT analysis was conducted on the pure EXAFS spectra presented in Figure 2c,d. The results, focusing on the *k* (0–12 Å^–1^) and *R* (2.5–4.5 Å) ranges, are summarized in Figure 3. Importantly, the full Δ*k* and Δ*R* ranges are not shown here: the corresponding full maps can be found instead in the Supporting Information, Section S4. Indeed, the lower values primarily highlight contributions from lighter atoms (e.g., oxygen from the framework and possibly adsorbed molecules), and no ambiguity exists in the attribution of the relative scattering contribution to either lighter or heavier atomic neighbors. Moreover, in the low-R range, the WT maps are known to be severely broadened along the k direction, thus carrying limited information on the elemental nature of the neighboring atoms.^40,51^ For this reason, only the portions of the WT maps at higher R-values are magnified and presented to better emphasize the more relevant and informative features.

The intensity maps reveal primarily two sublobes, present in all the Cu^II^ species. The first sublobe, located at lower *k*-values, corresponds to contributions from light atoms, as previously discussed. The second sublobe, at high-*k* values, is particularly informative as it represents the SS contribution from heavier atoms. Its position at around 7 Å^–1^ aligns with the maximum of the Cu–Cu or Cu–Zr backscattering function (see Supporting Information, Section S5).^40^ Given the nature of the system and the unlikelihood of Cu dimers forming within the MOF pores, the more plausible interpretation is that this sublobe arises from Cu–Zr SS, further supporting our earlier hypothesis regarding the local geometry of the Cu^II^ species. For **Cu**^**II**^**-HC** and **LC**, the sublobe profiles are comparable, suggesting similar local geometries for these sites. However, those associated with **Cu**^**II**^**-his** species appear more structured, with higher intensities in the low-k region. This likely reflects the presence of a greater number of light atoms, probably due to carbon atoms within the histidine unit. In the high-k region for the **Cu**^**I**^ component, no well-defined sublobe around 7 Å^–1^ is detected, likely due to an antiphase effect resulting from the structural disorder. To further understand the emerging structural complexity, we employed DFT-assisted EXAFS-fitting analysis on the MCR-derived pure components.

### 3.2 Computational Study

#### 3.2.1 Overview of Proposed Cu^II^ and Cu^I^ Species

**Figure 4:**
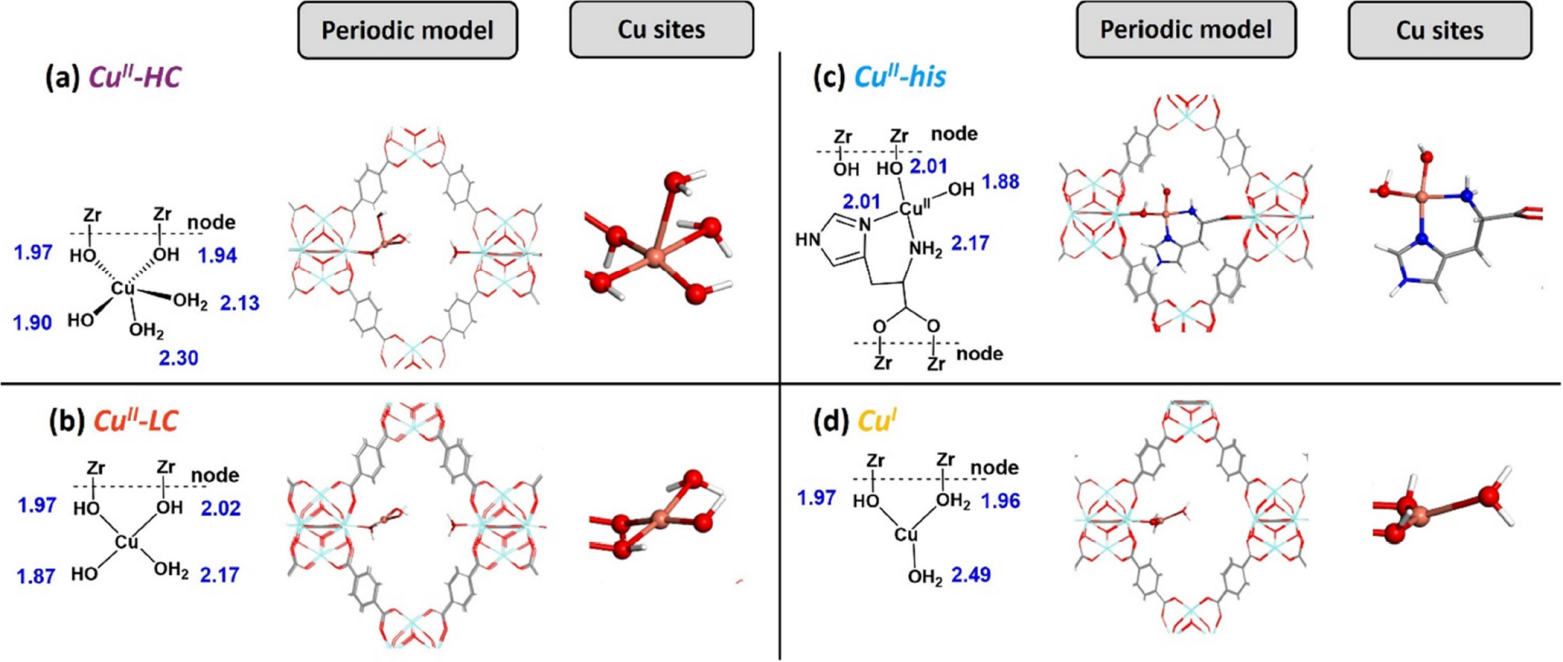
Representation of Cu sites in the histidine-modified Cu-UiO-66 obtained in this and previous studies.^26^ (a) Cu^II^-HC, (b) Cu^II^-LC, (c) Cu^II^-his and (d) Cu^I^. Bond distances were labeled in blue. Unpublished Figure, reporting data previously published in ref (26).

In our previous work,^26^ two Cu^II^ species were considered using periodic models: **Cu**^**II**^**-HC** and **Cu**^**II**^**-his** (Figure 4a,c). In this work, the same strategy was used to build the **Cu**^**II**^**-LC** system and **Cu**^**I**^ analogues (Figure 4b,d and Supporting Information, Figures S13 and S14). In the **Cu**^**II**^**-HC** structure, Cu is coordinated by three hydroxo and two water ligands. Upon dehydration, the tetra-coordinated Cu in **Cu**^**II**^**-LC** adopts a square planar configuration with Cu–OH bond distances at 1.87, 1.97, and 2.02 Å, and a Cu–H_2_O distance of 2.17 Å. In addition, two Cu^I^ structures were constructed involving tricoordinated Cu species bonded to either OH and OH_2_, with Cu–O distances of 1.96, 1.97, and 2.49 Å in **Cu**^**I**^, or OH and His, with a Cu–O distance of 1.96 and Cu–N distances of 2.01 and 2.10 Å in **Cu**^**I**^**-his** (see Figure S16).

#### 3.2.2 H_2_-Assisted Reduction Intermediates

**Figure 5:**
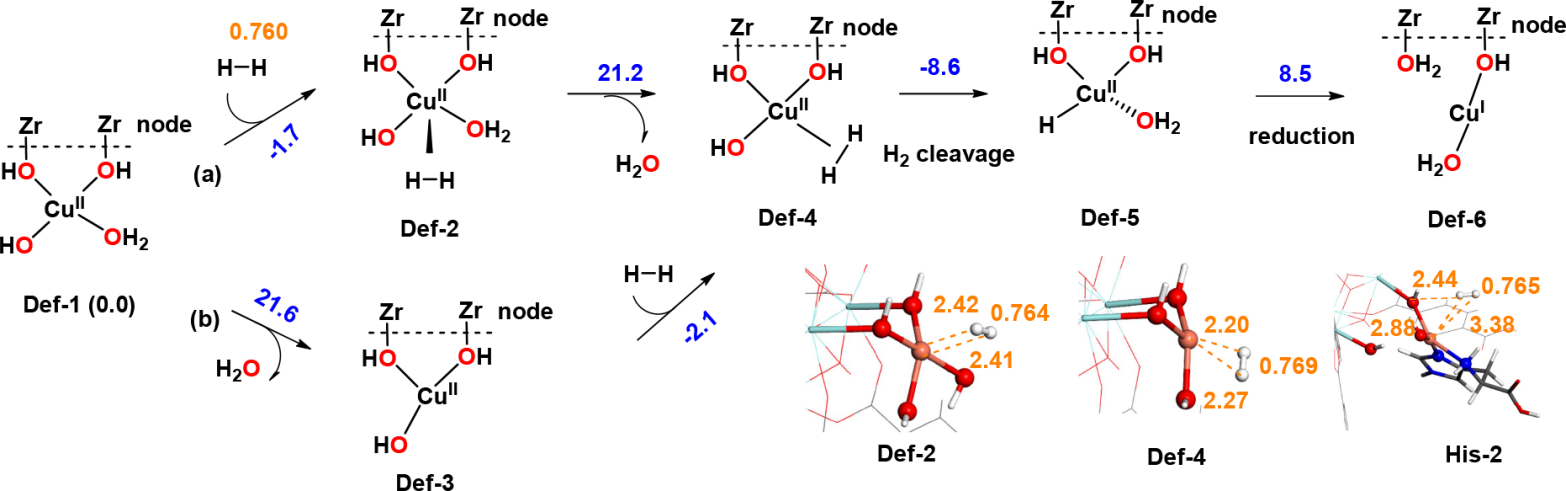
Reaction steps proposed for the Cu^II^-LC reduction using H_2_. Potential energy changes (in kcal mol^–1^) using cluster models are labeled in blue. Relevant interatomic distances, in Å, are labeled in orange.

To gain a deeper insight into the formation of the MCR-derived pure Cu^I^ species, the reaction intermediates involved during the reduction of Cu^II^-MOFs to Cu^I^-MOFs by H_2_ were investigated (Figure 5). The H_2_ dissociation on a supported metal may proceed through either homolytic or heterolytic cleavage pathways. Aireddy’s study^69^ indicated that the homolytic dissociation of H_2_ is generally more favorable than heterolytic dissociation on metal surfaces, except for Cu. Moreover, heterolytic dissociation can occur in the presence of a Lewis acid–base pair, such as the Cu^II^–O bonds in this work, preventing going through Cu^IV^ species. Therefore, only the heterolytic cleavage of H_2_ at Cu sites was considered.

Starting from the tetradentate Cu at the defective site **Def-1**, the H_2_ adsorption was considered by associative (via **Def-2**) and dissociative (via **Def-3**) ligand substitution pathways. The coordination of H_2_ to the tetra-coordinated Cu-site is exothermic by 1.7 kcal mol^–1^, while the dissociation of water is endothermic by 21.6 kcal mol^–1^. Despite this difference, the entropic contribution and irreversible water dissociation expected at 150 °C probably favor the dissociative over the associative pathway. The following steps are the coordination of H_2_ to **Def-3** and the heterolytic cleavage, which are thermodynamically favorable by 10.7 kcal mol^–1^. For the Cu reduction, we considered the transfer of electrons from the hydride to the rest of the framework, leading to the protonation of the OH ligands. Analysis of the spin density in **Def-6** shows that the unpaired electron has been transferred from Cu^II^ in **Def-5** to one of the aromatic rings of the linker (Figure S10), which also indicates the reduction of Cu^II^ to Cu^I^. This transformation was found to be endothermic by 8.5 kcal mol^–1^. A similar energy for the reduction step (11.7 kcal mol^–1^) was found using the periodic model. Despite this energy, the Cu^I^ structure using the periodic model showed an excellent fit with the experimental EXAFS results. This could be explained by an irreversible Cu^II^ to Cu^I^ reduction that could be caused by additional electron transfer from the linker to other species in the framework, such as free histidine or other Cu^II^ atoms.

Interestingly, we found that the reduction process from Cu^II^ to Cu^I^ was exothermic when considering the **Cu**^**II**^**-his** system by −8 kcal mol^–1^ (Figure S11 and Supporting Information, Section S6). However, this reduction process does not seem to occur experimentally. Indeed, the MCR-derived concentration profiles for this species tend to remain constant throughout the adopted experimental protocol. One possible explanation is the low affinity for H_2_ of the **Cu**^**II**^**-his** system. Indeed, we could not find any intermediate where H_2_ was adsorbed to the Cu^II^ attached to His, only a weak interaction of H_2_ with the bridged OH group as represented in Figure 5, with a Cu–H interatomic distances of 2.88 and 3.38 Å. These distances are significantly shorter in **Def-4** (Cu–H of 2.20 and 2.27 Å), where the H_2_ is clearly coordinated to Cu^II^. Another result supporting the inertness of the Cu-His sites is the bad match between the reduced Cu^I^–His with EXAFS results (see Supporting Information, Section 7.3).

### 3.3 DFT-Assisted Assignment of Pure Cu Species

#### 3.3.1 EXAFS-Fitting Results

Integrating qualitative and quantitative XAS findings with DFT-assisted EXAFS fitting provided successful results in discerning the possible MCR-derived pure Cu components present in both samples during the experiment. The parametrization of scattering paths involved fixing the passive amplitude reduction factor (S_0_^2^) at 1.0, the ideal value for this parameter.^72^ Simultaneously, the same energy shift (Δ*E*) and all the SS parameters were refined during the analysis for all the included scattering paths. For further details on the specific method and on the adopted parametrization, the reader can refer to the Supporting Information, Section 7.1.

**Figure 6:**
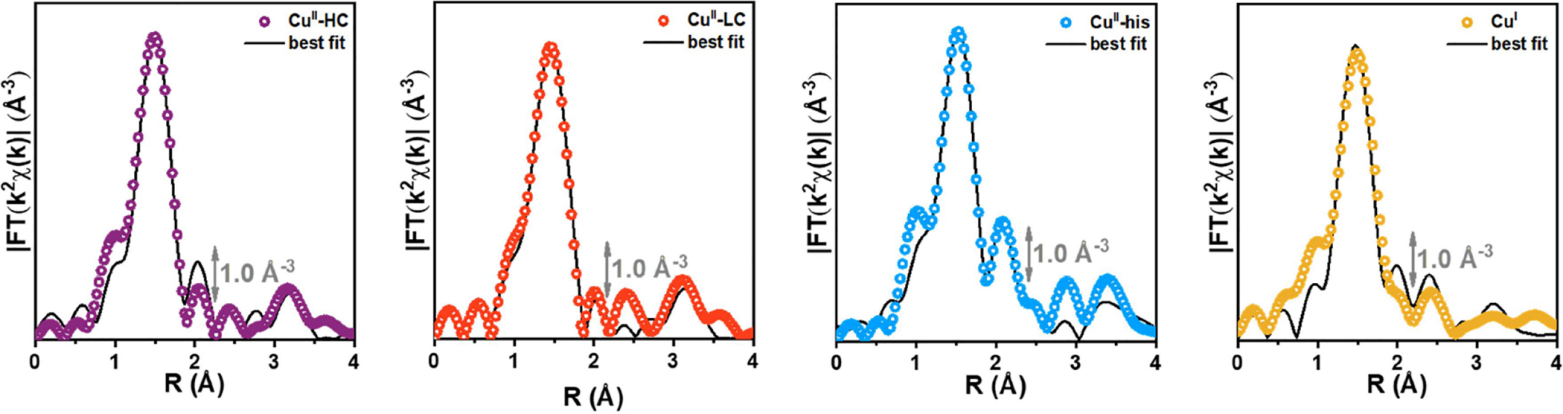
Phase-uncorrected modulus of experimental and best fit FT-EXAFS spectra for the MCR-derived pure species.

Guided by the qualitative and quantitative spectroscopic findings discussed so far, a series of DFT-optimized structures was designed for the four Cu-species identified. The FT-EXAFS spectra shown in Figure 6 are accurately reproduced by the best-fit curves, with the agreement supported by low R-factors and physically meaningful parameter values (Table 1). In all cases studied, the peak corresponding to the first coordination sphere is perfectly reproduced by the proposed structures. The penta-coordinate Cu site within the defective Zr-cluster (**Cu**^**II**^**-HC**), as suggested by Aunan et al.,^26^ is here confirmed to be one of the initial motifs in the system, well-represented by a more symmetric site with an average bond distance of (1.941 ± 0.009) Å. The Cu coordination environment is completed by a more distant H_2_O extra-ligand molecule at (2.24 ± 0.03) Å.

**Table 1:** EXAFS-Fitting Results Obtained Performing the Analysis on the *k*^2^-Weighted FT-EXAFS Experimental Spectrum of the MCR-Derived Pure Cu Species^a^

EXAFS parameters	Cu^II^-HC	Cu^II^-LC	Cu^II^-his	Cu^I^
*N*°_par_/*N*°_ind_	8/16	7/15	9/17	9/14
*R*_factor_	0.02	0.02	0.03	0.04
Coord. Num.	5	4	4	3
S_0_^2^	1.0	1.0	1.0	1.0
Δ*E* (eV)	0 **±** 1	1.0 ± 0.1	–7 ± 2	2 ± 1
R_O_1_ (Å)	1.94 ± 0.01	1.93 ± 0.09		1.85 ± 0.02
R_O_2_ (Å)	2.22 ± 0.03			2.76 ± 0.05
R_N,O (Å)			1.98 ± 0.01	
R_C (Å)			2.74 ± 0.09	
R_Zr (Å)	3.75 ± 0.02	3.67 ± 0.04	4.0 ± 0.1	3.48 ± 0.1
σ^2^_O_1_ (Å^2^)	0.004 ± 0.001	0.007 ± 0.002		0.002 ± 0.001
σ^2^_O_2_ (Å^2^)	0.004 ± 0.002			0.001 ± 0.004
σ^2^_N,O (Å^2^)			0.001 ± 0.001	
σ^2^_C (Å^2^)			0.01 ± 0.01	
σ^2^_Zr (Å^2^)	0.008 ± 0.01	0.010 ± 0.005	0.005 ± 0.002	0.01 ± 0.01

a The parameters set in the analysis are underlined.

The loss of a water molecule during the initial heating step in inert gas flow was proposed to result in the **Cu**^**II**^**-LC** species, a tetra-coordinated Cu^II^ site which maintains a symmetric first coordination shell with an average Cu–O distance of (1.93 ± 0.09) Å. Subsequently, a 3-fold T-shaped **Cu**^**I**^ site anchored to the defective Zr-cluster was proposed, falling in line with the MCR reconstruction reported in the previous section. Regarding the **Cu**^**I**^ site, the optimized average bond distance was found to be shorter (1.83 ± 0.02 Å), which is consistent with changes associated with the oxidation state.

The **Cu**^**II**^**-his** structure reflects the model proposed by Aunan et al., with a Cu^II^ site symmetrically coordinated to both the defective cluster and the histidine unit, exhibiting an average bond distance of (2.00 ± 0.04) Å, and playing no specific role during the experimental procedure. To further validate this hypothesis, EXAFS fitting analysis was performed on a proposed Cu^I^-his structure, as detailed in the Supporting Information, Section 7.3. However, the resulting values were not physically reliable and were subsequently discarded.

Upon closer examination, for the **Cu**^**II**^**-HC**, **Cu**^**II**^**-LC,** and **Cu**^**I**^ species, the second shell signals are caused by the SS contributions of O atoms within the Zr node, consistent with the preferential location of these Cu species on the defective node site. Conversely, the second-shell signal for the **Cu**^**II**^**-his** is primarily attributed to C atoms within the histidine unit, suggesting that the Cu site is located further from the MOF node. This hypothesis is supported by the analysis of the third coordination shell conducted by Aunan et al., which is not fully reproduced, as in the other Cu^II^ cases (at ca. 3.50 Å), due to the absence of SS contribution from the Zr atom. In general, for all the studied cases, the reproduction of further signals is satisfactory but not entirely accurate. This is likely due to the omission of multiple scattering (MS) paths from the analysis, making it challenging to detect scattering contributions from atoms located at distances greater than 3.5 Å.

According to these fitting results, the experimental data rigorously validate our qualitative and quantitative hypotheses on the basis of the pure MCR-derived Cu components found in both the samples.

#### 3.3.2 XANES Simulation Results

**Figure 7:**
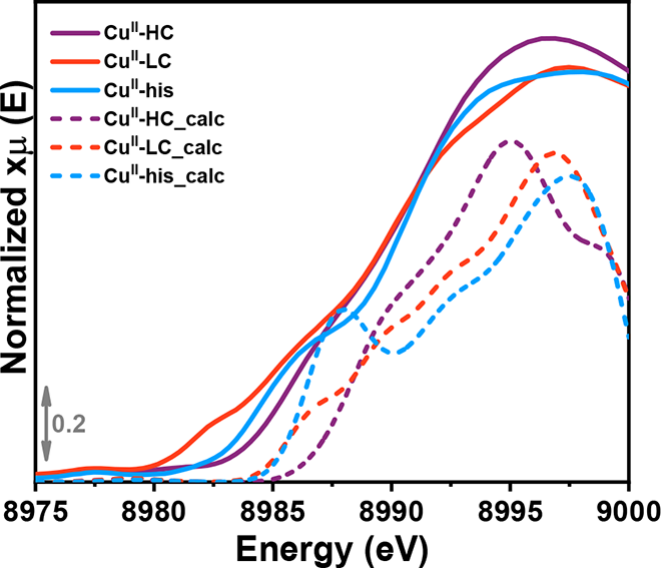
MCR-derived (solid lines) and calculated (dashed lines) XANES spectra related to the identified Cu^II^ species.

As discussed in the previous sections, MCR analysis revealed the presence of four distinct Cu pure species. While the **Cu**^**I**^ spectrum is clearly distinguishable, the Cu^II^ species exhibit similar spectroscopic features. To strengthen the structural motifs proposed earlier, we employed DFT-assisted XANES simulation to reproduce the Cu^II^ features in their respective pure XANES spectra. The calculated rising-edge XAS of **Cu**^**II**^**-HC**, **Cu**^**II**^**-LC**, and **Cu**^**II**^**-his** are shown in Figure 7. The signal observed at around 8985.8 eV in both **Cu**^**II**^**-LC** and **Cu**^**II**^**-his** corresponds to the 1s → 4p electronic transition of Cu^II^ and is accurately reproduced in the respective calculated spectra. Moreover, the signal is less pronounced in **Cu**^**II**^**-LC**, suggesting significant differences in the Cu first-shell coordination geometry compared to **Cu**^**II**^**-his**. In contrast, no peak is observed at this energy position for **Cu**^**II**^**-HC**, supporting our previous hypothesis regarding the structural interpretation of the Cu^II^ sites present in our samples. The spectrum of **Cu**^**I**^ species was not considered due to the cluster models poorly replicating the geometry of the periodic model (see more details in Supporting Information, Section 7.4).

## 4 Discussion

This study aimed to advance the understanding of the chemical nature and speciation of Cu species formed in two distinct bioinspired Cu-UiO-66 MOF samples during a model redox experiment through the integration of advanced spectroscopy, data analysis, and DFT calculations. The Cu site coordinated to histidine molecules, as observed in LPMO enzymes, has been shown to be an active motif for the oxidation of C–H bonds in light alkanes.^27,71^ By emulating and probing the behavior of this catalytic center in a model redox experiment, we aim to unlock novel insights into this pivotal reaction. In this section, we critically discuss the obtained results, considering previous literature and emphasizing how the innovative methodologies implemented in this work contribute to the atomic-scale understanding of Cu-MOF-based materials.

**Scheme 1:**
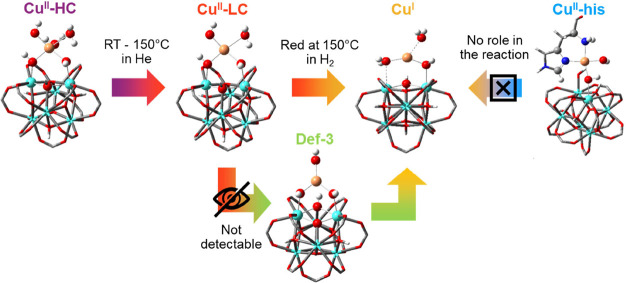
Pictorial representation of the proposed redox chemistry during the experiment, connecting the magnified DFT-calculated Cu sites.

Building upon previous research by Aunan et al. on the same systems,^26^ we initially characterized the Cu species in the as-prepared samples, identifying two distinct motifs: a Cu^II^ atom residing at a Zr-defective site (**Cu**^**II**^**-HC**) and Cu^II^ coordinated both to the defect and a histidine molecule (**Cu**^**II**^**-his**). The application of MCR-ALS analysis allowed us to quantitatively assess the abundance of these two species in the as-prepared samples, which aligned remarkably well with the postsynthesis elemental analysis. Furthermore, MCR-ALS enabled us to track the evolution of other pure Cu species present in the mixture during the in situ experiment. A key advancement of this study was the novel application of EXAFS fitting on the MCR-derived species, which proved crucial for elucidating the structural properties of the pure Cu sites. Notably, the Cu species localized at the defective Zr cluster exhibited a distinctive redox chemistry and enhanced reactivity under the adopted experimental conditions, as depicted in Scheme 1. This observation suggests that structural defects within the MOF framework play a pivotal role in facilitating electron transfer processes, thereby boosting the redox activity of the associated Cu species. This finding is consistent with prior studies, which have highlighted the catalytic benefits of MOF defects, but our work provides direct structural evidence supporting this behavior. Interestingly, the Cu species coordinated to histidine displayed partial inertness, with samples containing a higher histidine content exhibiting reduced activity in the redox reaction. This result was attributed to the lower H_2_ affinity of the tetracoordinated Cu^II^ sites attached to His compared to those attached to the Zr-nodes, which can release water molecules leading to highly unsaturated Cu-sites such as **Def-3** in Figure 5.

While this study primarily focused on elucidating the reduction mechanism of the Cu sites in the H_2_ model feed, the reoxidation process was also comparatively characterized, by exposing the reduced sample state to O_2_, working isothermally at 150 °C. Our MCR-ALS analysis did not yield additional distinct Cu^II^ species during the oxidation phase and attempts to increase the principal components (PCs) to five resulted in unreliable reconstructions due to the similarity of the species in the mixture. Based on this, we propose that the oxidation step predominantly regenerates the initial species, specifically **Cu**^**II**^**-HC** and **Cu**^**II**^**-LC**. The effective restoration of the more highly coordinated Cu^II^ species upon exposure to O_2_ suggests the presence of trace water molecules, which may facilitate the oxidation by coordinating to the Cu^I^ site. Nonetheless, we could not completely rule out the presence of other O_2_-derived Cu^II^ species, possibly forming in limited abundance and/or featuring spectral features largely overlapped with those of the **Cu**^**II**^**-HC** and **Cu**^**II**^**-LC** moieties. With this respect, future studies employing high energy resolution fluorescence detected (HERFD-)XANES could be envisaged, attempting to enhance the resolving power of the technique toward structurally similar Cu^II^ species.^73,74^

These findings provide valuable insights into the redox chemistry of Cu species in MOFs and the intricate relationship between their structure and reactivity. Moreover, this study offers a detailed characterization of Cu redox processes, which could inform future research on other important reactions, such as methane-to-methanol (MTM) conversion, where the Cu^II^/Cu^I^ redox cycle plays a critical role.

## 5 Conclusions

This study provides detailed insights into the structural dynamics of copper speciation in histidine-modified Cu-UiO-66 MOFs. We collected in situ XAS spectra from two compositionally distinct MOFs during in situ experiments under model redox conditions, utilizing H_2_ as the reducing agent and O_2_ as the oxidizing agent at 150 °C.

Using MCR-ALS analysis, implemented via the *Waterfall approach*, we identified and characterized four distinct pure Cu species forming inside the MOF scaffold during the employed model red-ox protocol. These species were defined in accordance with the initial hypotheses presented by Aunan et al. in a previous work focused on the same type of histidine-modified Cu-UiO-66 MOFs in their as-prepared state. The experimental conditions facilitated the application of DFT-assisted EXAFS fitting analysis on the MCR-derived pure species, representing a novel approach to achieve a comprehensive characterization of the evolving Cu sites within the mixture.

Furthermore, advanced data analysis techniques, including WT analysis on the pure EXAFS spectra, were crucial in assessing the Cu–Zr single scattering contributions across all Cu^II^ species, thus corroborating our structural hypotheses. The clear identification of a chemical reaction involving Cu sites within the defective Zr cluster further elucidates the accessibility and limitations of bioinspired systems, enhancing our understanding of these materials. Finally, the novel methodologies introduced in this work can be extended to various other reactions, particularly those conducted in mild conditions (<200 °C) with limited temperature swaps, which guarantee higher quality and reliability of the MCR-derived EXAFS, limiting the impact from the Debye–Waller factor variations, and thereby simplifying the analysis and interpretation of structural changes.

## Supporting Information Available

Additional information on compositional and physicochemical properties of samples; details on XAS MCR-ALS reconstruction and WT-EXAFS analysis; additional details on the Cu^II^ reduction by H_2_; thermodynamics calculated by the PBE functional; spin density on the Cu^I^ at the defective sites; thermodynamics on Cu^II^ reduction at the histidine sites; additional EXAFS fitting results; comparison of geometries with periodic and cluster models (PDF)
