# Supplementary material for: Pinpointing Cu-Coordination Motifs in Bio-Inspired MOFs by Combining DFT-Assisted XAS Analysis and Multivariate Curve Resolution
Source: J Phys Chem C Nanomater Interfaces. 2025 Feb 6;129(7):3570–82. doi: 10.1021/acs.jpcc.4c08029 (PMC11848919; doi:10.1021/acs.jpcc.4c08029)
Supplement: Supplementary file 1 — jp4c08029_si_001.pdf [file jp4c08029_si_001.pdf]

PINPOINTING CU-COORDINATION MOTIFS IN BIO-INSPIRED MOFS BY COMBINING DFT-ASSISTED XAS ANALYSIS AND MULTIVARIATE CURVE RESOLUTION

Beatrice Garetto[a†], Ning Cao[b,c†], Valeria Finelli[a,d], Erlend Aunan[b], Matteo Signorile[a], Unni Olsbye[b], Silvia Bordiga[a], Ainara Nova[b,c*], Elisa Borfecchia[a*]

[a]Department of Chemistry, NIS and INSTM Reference Centre, Università di Torino, Via G. Quarello 15/A, I-10135, and Via P. Giuria 7, I-10125, Turin, Italy

[b]SMN Centre for Material Science and Nanotechnology, Department of Chemistry, University of Oslo, N-0315, Oslo, Norway

[c]Hylleraas Centre for Quantum Molecular Sciences, Department of Chemistry, University of Oslo, P. O. Box 1033, Blindern, N-0315, Oslo, Norway

[d]University School of Advanced Studies, IUSS Pavia, Palazzo del Broletto, P.zza della Vittoria 15, I-27100, Pavia, Italy

[†]The authors equally contributed.

Table of Contents

1 Compositional and physico-chemical properties of samples 2

2 Additional Infrared (IR) and Diffraction (PXRD) analysis 2

2.1 PXRD results 2

2.2 In situ FT-IR results 3

3 Additional details on XAS analysis 4

3.1 MCR-ALS quality indicators 4

3.2 Energy references for XANES simulation alignment 4

4 Additional details on EXAFS Wavelet Transform Analysis 4

5 F(k) curves for Cu-Cu and Cu-Zr scattering paths 7

6 Additional details on the Cu(II) reduction by H_2 8

6.1 Thermodynamics calculated by the PBE functional 8

6.2 Changes in spin density and charge population 8

6.3 Thermodynamics on Cu^{II} reduction at the histidine sites 9

7 Additional details on EXAFS fitting 9

7.1 Parametrization details 9

7.2 Detailed EXAFS fitting results for MCR-derived Cu-species 10

7.3 Additional results on EXAFS fitting for Cu^{I}-his 11

7.4 Comparison of geometries with periodic and cluster models 13

8 References 13

1 Compositional and physico-chemical properties of samples

Table S1. Compositional and physico-chemical properties of the investigated MOF samples. [1] Unpublished Table, reporting data previously published in [1].

Name	Sample	Estimated composition per $[Zr_6O_4(OH)_4]^{12+}$					Cu:his	Surface area¤	Pore volume§
		BDC	Oac	$[OH+H_2O]^-$	His	Cu		$[m^2g^{-1}]$	$[cm^3g^{-1}]$
UiO-66	Defective UiO-66	4.6	0.5	2.3	-	-	-	1694	0.67
His-1	UiO-66-$his_{0.31}$	4.3	0	3.1	0.31	-	-	1563	0.65
His-2	UiO-66-$his_{0.08}$	4.3	0.1	3.3	0.08	-	-	1677	0.68
His-Cu-1	UiO-66-$his_{0.31}Cu_{0.81}$	4.3	0	3.1	0.31	0.81	2.6	1507	0.64
His-Cu-2	UiO-66-$his_{0.08}Cu_{0.56}$	4.3	0.1	3.3	0.08	0.56	7	1725	0.71

¤: Specific suface area as measured with BET, using N_2 adsorption at 77K.
§: Pore volume measured at p/p_0=0.8, using N_2 adsorption at 77K.

2 Additional Infrared (IR) and Diffraction (PXRD) analysis

2.1 PXRD results

Powder X-Ray Diffraction (PXRD) patterns of the MOFs were collected on a PANalytical X'Pert instrument with Cu Kα radiation corresponding to an incident wavelength λ = 1.5405 Å. The patterns were acquired in the 2θ range of 3-30° with a step size of 0.02°, using the Bragg-Brentano geometry. PXRD patterns (Figure S1) were collected before and after the experiment to check the structural integrity of the pelletized samples re-exposed to air at the end of the spectroscopic experiments. The crystallinity is preserved in all the reported cases.

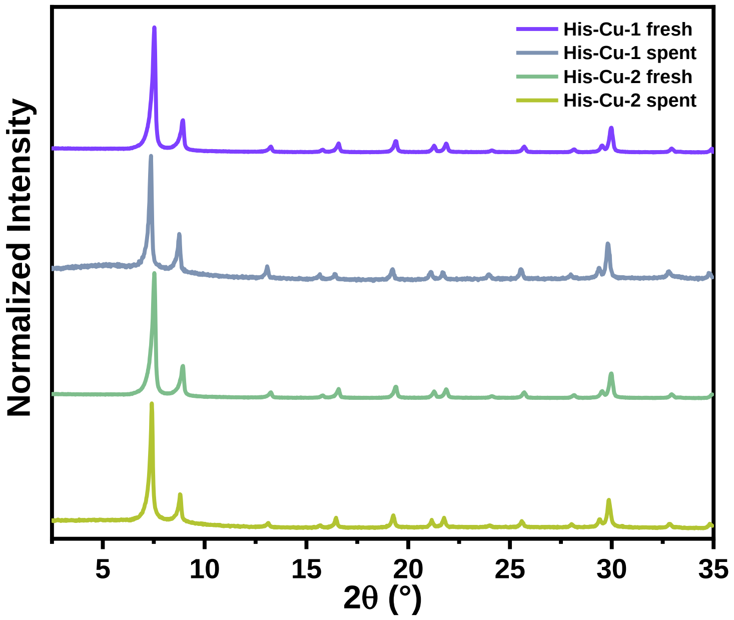

Figure S1: Powder diffraction pattern for both the samples, collected before (fresh) and after (spent) the experiment.

2.2 In situ FT-IR results

In situ FT-IR spectra were collected with a resolution of 2 cm^{-1} by averaging 32 scans in transmission mode using an AABSpec A1000 cell[2] placed in a Bruker INVENIO R FT-IR spectrometer, equipped with a Mercury-Cadmium-Telluride (MCT) cryogenic detector. Prior to the transmission IR measurements, the samples were pelletized at a pressure of 0.5 ton: the use of higher pressures was avoided to prevent (partial) sample amorphization. The self-supported pellets were then placed in the sample holder. This apparatus was coupled to a gas feeding system equipped with Bronkhorst El-Flow digital mass flow controllers for samples activation under He flow (25 mL/min) from RT up to 150 °C (heating ramp 3 °C/min). After IR measurements, PXRD patterns were collected to check the structural integrity of the pelletized samples re-exposed to air at the end of the spectroscopic experiments.

Figure S2 displays the spectral evolution for the activation of His-Cu-1 under He flow (25 mL/min) with heating ramp 3 °C/min up to 150 °C, isothermally treating the material at 150 °C for 1 h. The same activation protocol was adopted for His-Cu-2, as displayed in Figure S3. The spectra of the treated MOFs are dominated by the carboxylates vibrations (bands in the range 1640-1215 cm^{-1} out of scale) and by an intense band at 3674 cm^{-1}, tailed on the low-frequency side, associated to the stretching of isolated μ3–OH species on the cornerstones of the Zr_6-clusters of the MOF.[1] A fine spectroscopic characterization of these materials is already present in literature.[1]

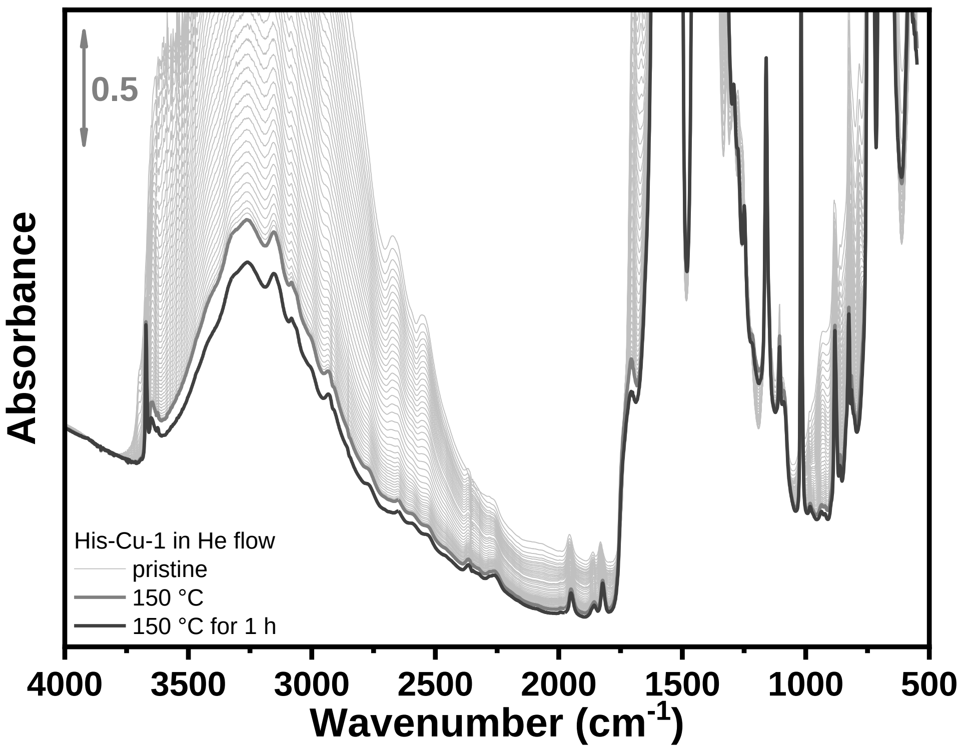

Figure S2: in situ IR spectra of His-Cu-1 for monitoring its solvent removal in He flow.

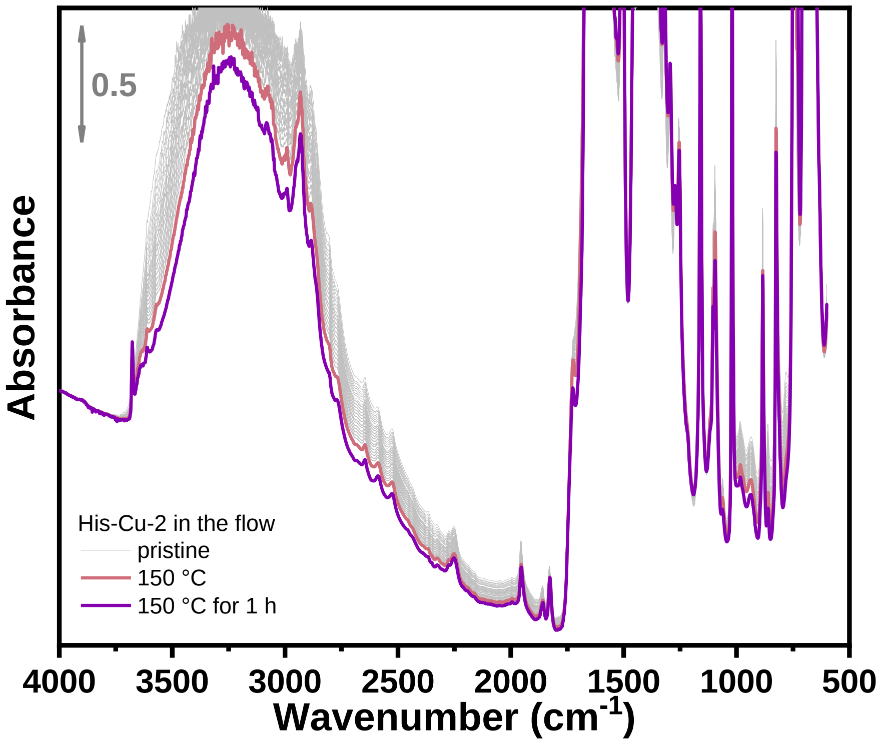

Figure S3: in situ IR spectra of His-Cu-2 for monitoring its solvent removal in He flow.

3 Additional details on XAS analysis

3.1 MCR-ALS quality indicators

Table S2. Quality indicators of the MCR-ALS analysis (PCs = 4) of the in situ XAS dataset collected during the experiment.

Quality indicators	Value
Std. Deviation of residual vs exp. Data	0.0021684
Fitting error (LoF) of PCA	0.0621195
Fitting error (LoF) of exp.	0.19873
Iteration number to the convergence	47
Percentage of variance explained at the optimum	99.9994

3.2 Energy references for XANES simulation alignment

Table S3. Calibration on pre-edge positions between experimental and calculated XAS

	Exp. Pre-edge position	Cal. Pre-edge position	Calibration
[Ref64]	8979.5 eV	8982.46 eV	-2.96 eV
[Ref65]	8979.5 eV	8982.27 eV	-2.77 eV
[Ref66]	8979.9 eV	8982.71 eV	-2.81 eV
		Average Calibration	-2.9 eV

4 Additional details on EXAFS Wavelet Transform Analysis

Full-range Wavelet Transform (WT) maps are reported hereafter (Figure S4-S7). As also explained in the main text, the lower k and R values primarily highlight contributions from lighter atom (e.g. oxygen from

framework and extra framework adsorbed molecules), and no ambiguity exist in the attribution of the relative scattering contributions to either lighter or heavier atomic neighbours. For the sake of clarity, both the full range and the magnification are reported for all the MCR-derived pure Cu species.

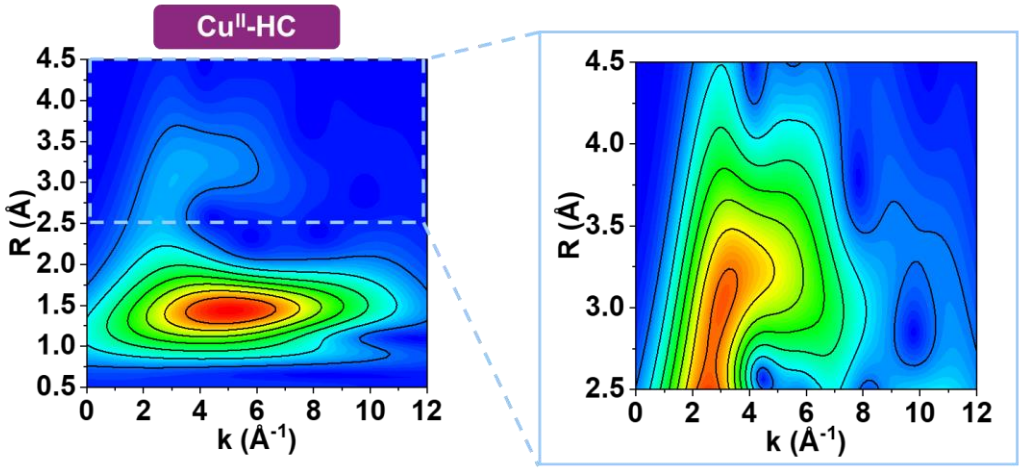

*Figure S4. Left) Full-range WT representation of the EXAFS signal for the **Cu^{II}-HC** MCR-derived pure species. Right) Magnification of the high-R WT region highlighted by the light blue dashed box, in the ranges Δk (0–12) Å^{-1} and ΔR (2.5–4.5) Å.*

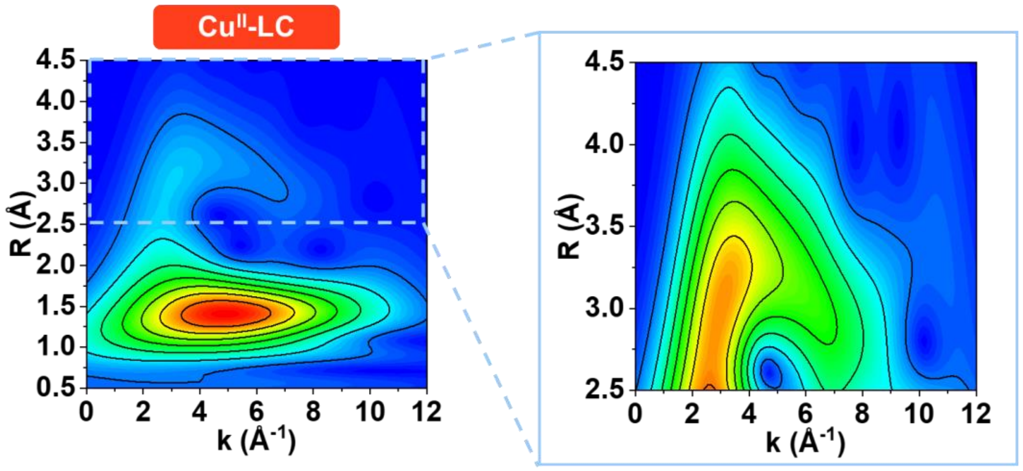

*Figure S5. Left) Full-range WT representation of the EXAFS signal for the **Cu^{II}-LC** MCR-derived pure species. Right) Magnification of the high-R WT region highlighted by the light blue dashed box, in the ranges Δk (0–12) Å^{-1} and ΔR (2.5–4.5) Å.*

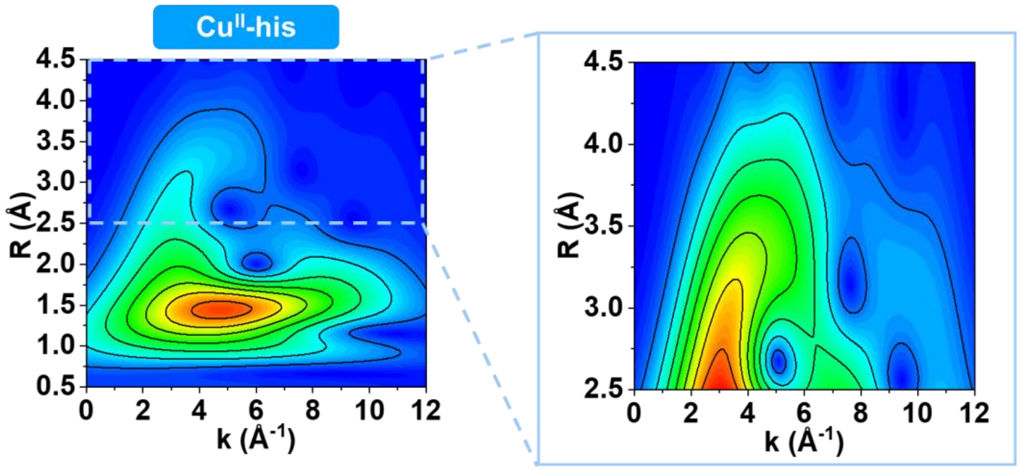

*Figure S6. Left) Full-range WT representation of the EXAFS signal for the **Cu^{II}-his** MCR-derived Cu-species. Right) Magnification of the high-R WT region highlighted by the light blue dashed box, in the ranges Δk (0–12) Å^{-1} and ΔR (2.5–4.5) Å.*

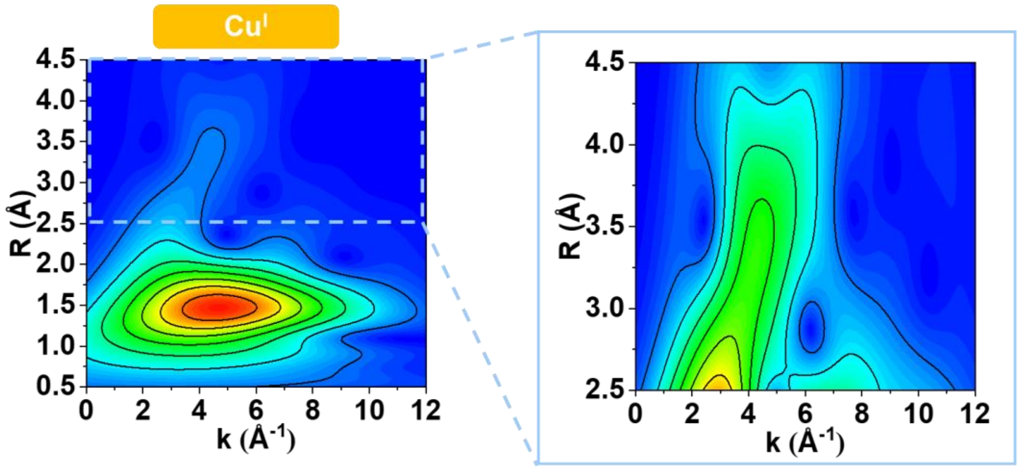

*Figure S7. Left) Full-range WT representation of the EXAFS signal for the **Cu^{I}** MCR-derived pure species. Right) Magnification of the high-R WT region highlighted by the light blue dashed box, in the ranges Δk (0–12) Å^{-1} and ΔR (2.5–4.5) Å.*

5 F(k) curves for Cu-Cu and Cu-Zr scattering paths

In general, in k-space, Cu–Cu or Cu–Zr single scattering paths can be discriminated from those involving framework atoms due to a different EXAFS backscattering amplitude function (F(k)) of the elements, leading to maxima of the F(k) function localized at different, element-specific, k-space values. Hence, to discriminate the signals present in the WT maps, is sufficient the comparison between the position of the F(k) curves and the sublobes, as demonstrated with an example in Figure S8.

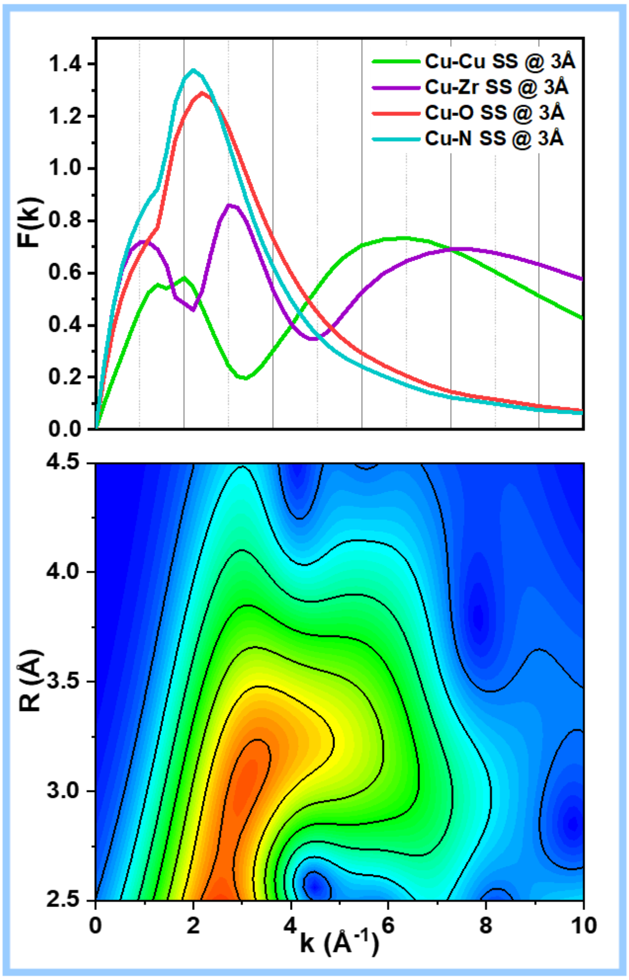

*Figure S8. Magnification of the high-R WT region in the ranges Δk (0–10) Å^{-1} and ΔR (2.5–4.5) Å related to the **Cu^{II}-HC** pure species. On the top is reported a plot of the relevant backscattering amplitude functions, F(k), calculated by FEFF6.0 considering SS paths involving O, N, Cu and Zr scatterers.* [3]

The second sub-lobe, at high-k values, is centred at around 7 Å^{-1}, which aligns the maximum of the Cu-Cu or Cu-Zr backscattering function. Given the nature of the system and the unlikelihood of Cu dimers forming within the MOF pores, the more plausible interpretation is that this sub-lobe arises from Cu-Zr SS in Cu-species localized in the proximity of the MOF nodes.

6 Additional details on the Cu(II) reduction by H_2

6.1 *Thermodynamics calculated by the PBE functional*

We observed significant geometry differences for the Cu^{I} structures using the cluster model with PBE0 functional and the periodic model with PBE (see Figure S18). To determine the origin of this difference, we also used the PBE functional to optimize the cluster models and compared the thermodynamics with the results obtained with the PBE0 functional. As shown in Figures 5 and S9, reaction thermodynamics involving Cu^{II} species are very similar, with differences lower than 1 kcal mol^{-1}. However, the reaction thermodynamic for the Cu reduction is significantly different with the two methods: 22.4 kcal/mol for the PBE functional and 8.5 kcal/mol for the PBE0 functional.

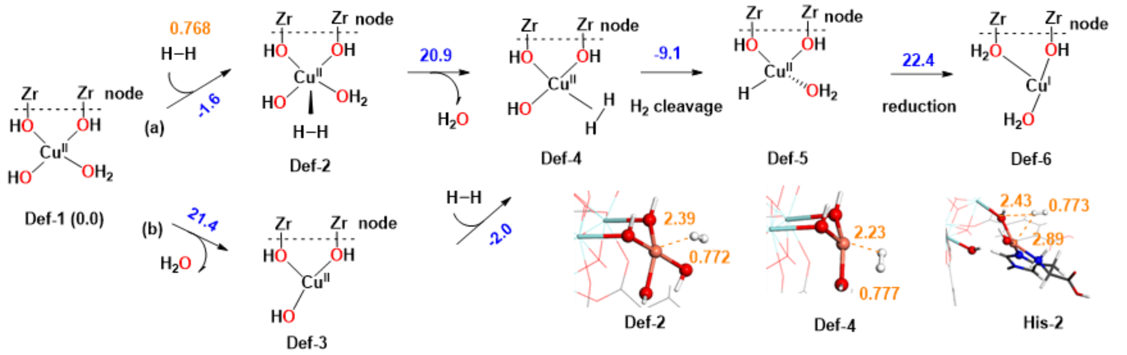

Figure S9. Reaction steps proposed for the Cu^{II}-LC reduction using H_2. Potential energy changes (in kcal mol^{-1}) using PBE and cluster models are labelled in blue. Relevant interatomic distances, in Å, are labelled in orange.

6.2 *Changes in spin density and charge population*

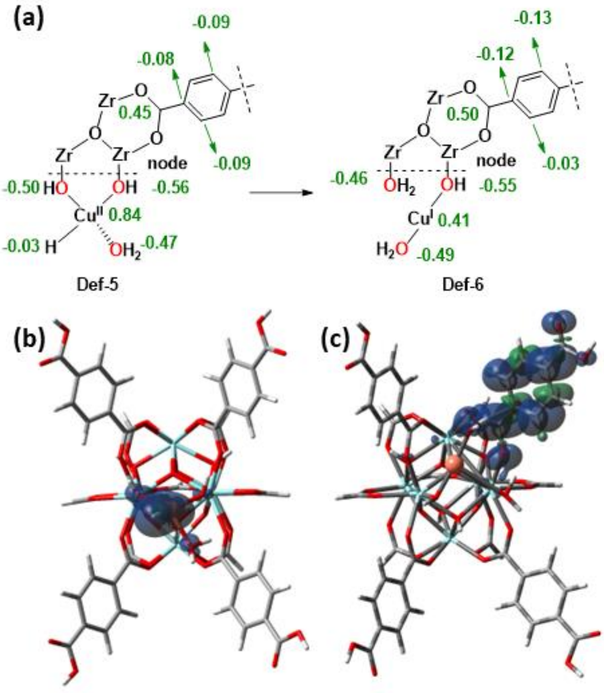

Figure S10.Change in charge populations (a) and spin densities from Def-5 (b) to Def-6 (c). The NPA charge analysis suggests a decrease of charge on Cu and an increase in the carbon atoms in the phenyl linker. The lack of spin density in Cu indicates that it has been reduced to Cu(I).

6.3 *Thermodynamics on Cu^{II} reduction at the histidine sites*

We also considered the heterolytic cleavage of H_2 on the histidine site (Figure S11). The reduction process resembles the one with Cu at the defective site. However, for this system, all reaction steps are exothermic, including the Cu^{II} to Cu^{I} reduction (**His-3** to **His-4**). Another difference is the long Cu-H interatomic distances of 2.88 Å compared with the O-H distances of 2.44 Å, indicating that H_2 is not adsorbed to Cu^{II} but interacts with the OH groups by hydrogen bonding.

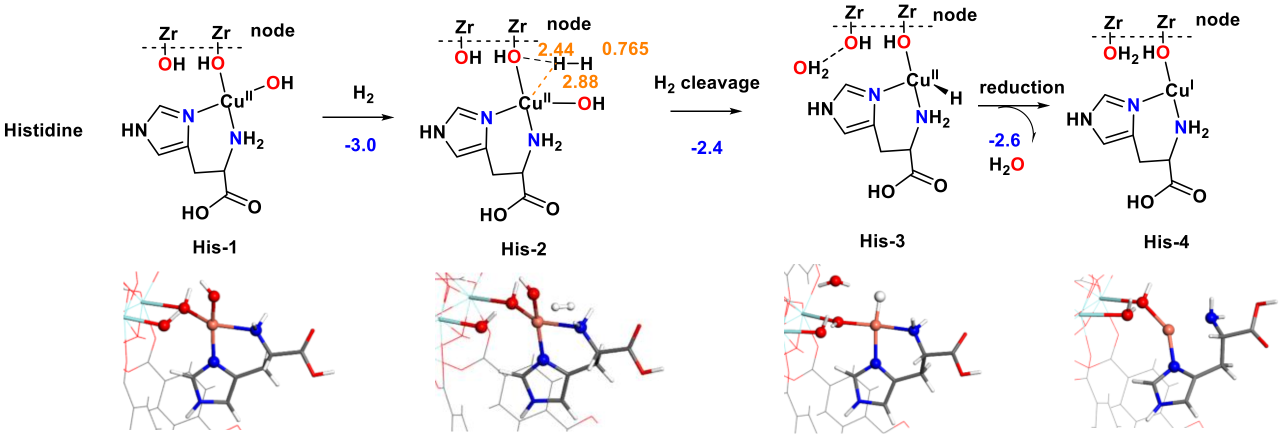

Figure S11. Reaction steps computed for the Cu^{II}-his reduction using H_2. Potential energy changes (in kcal mol-1) using cluster models are labelled in blue. Relevant interatomic distances, in Å, are labelled in orange.

7 Additional details on EXAFS fitting

7.1 *Parametrization details*

The EXAFS fitting analysis was performed on the pure Cu spectra derived from the MCR analysis. The fitting procedure was conducted in R-space within the 1.0-4.0 Å range for all the DFT-optimized models. This process is carried out by using the Artemis code from the Demeter suite,[4] on the k^2-weighted EXAFS spectra, Fourier transformed in the 2.5-11.0 $Å^{-1}$ range.

In all the cases (Figure S12-S15), the parametrization of scattering paths involved fixing the passive amplitude reduction factor (S_0^2) at 1.0,[5] the ideal value for this parameter. Simultaneously, the same energy shift (ΔE) was refined during the analysis for all the included scattering paths. For the Cu ions in the defective Zr node (**Cu^{II}-HC**, **Cu^{II}-LC** and **Cu^{I}**), the fit incorporated only SS paths, encompassing first-shell O atoms from hydroxyl extra-ligands and from Zr MOF node (O_1) species, as well as Zr atoms belonging to the cluster (Zr). The SS parametrization grouped atoms related to the same atom-type and coordination shell, which were parametrized using the same radial shift parameter (ΔR) and Debye-Waller factor (σ^2). Notably, a distinct ΔR and σ^2 was employed for the O atom associated with the extra-ligand H_2O molecule (denoted as O_2) located at ca. 2.3 Å from the Cu centre in the Cu^{II}-HC structural model, reflecting a more distant contribution. In the meantime, the four O atoms associated with OH-groups were combined in the same ΔR and σ^2 parametrization, assuming comparable interatomic distances at Reff = 1.95 Å from the Cu centre and, accordingly, a more symmetric local surrounding. The

SS path of two Zr atoms at average Reff = 3.75 Å were parameterized with ad hoc ΔR_{Zr} and σ^2_{Zr} parameters.

7.2 Detailed EXAFS fitting results for MCR-derived Cu-species

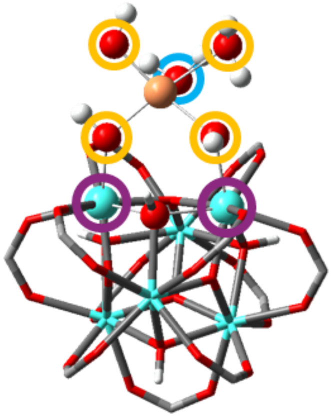

EXAFS parameters	Cu^{II}-HC
$N°_{par}/N°_{ind}$	8/16
R_{factor}	0.02
S_0^2	1.0
ΔE (eV)	0 ± 1
R_O_1 (Å)	1.94 ± 0.01
R_O_2 (Å)	2.22 ± 0.03
R_Zr (Å)	3.75 ± 0.02
$\sigma^2\_O_1$ (Å²)	0.004 ± 0.001
$\sigma^2\_O_2$ (Å²)	0.004 ± 0.002
σ^2_Zr (Å²)	0.01 ± 0.01

*Figure S12. Left) Pictorial representation of the DFT-optimized **Cu^{II}-HC** structure, in which the SS contributions are highlighted with coloured circles. Right) results of the fits performed on the k^2-weighted FT-EXAFS experimental spectrum of MCR-derived **Cu^{II}-HC** species.*

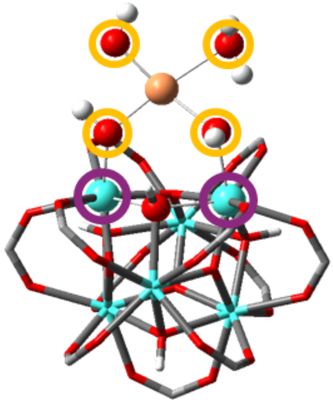

EXAFS parameters	Cu^{II}-LC
$N°_{par}/N°_{ind}$	**7/15**
R_{factor}	0.02
S_0^2	1.0
ΔE (eV)	1.0 ± 0.1
R_O_1 (Å)	1.93 ± 0.09
R_Zr (Å)	3.67 ± 0.04
$\sigma^2\_O_1$ (Å²)	0.007 ± 0.002
σ^2_Zr (Å²)	0.010 ± 0.005

*Figure S13. Left) Pictorial representation of the DFT-optimized **Cu^{II}-LC** structure, in which the SS contributions are highlighted with coloured circles. Right) results of the fits performed on the k^2-weighted FT-EXAFS experimental spectrum of MCR-derived **Cu^{II}-LC** species.*

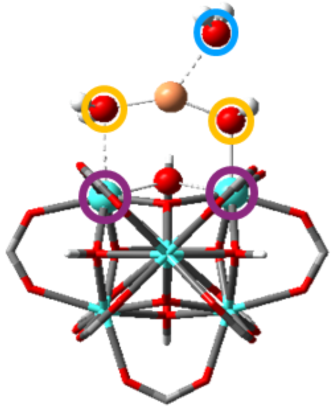

EXAFS parameters	Cu^{I}
$N°_{par}/N°_{ind}$	**9/14**
R_{factor}	0.04
S_0^2	1.0
ΔE (eV)	2 ± 1
R_O$_1$ (Å)	1.85 ± 0.02
R_O$_2$ (Å)	2.76 ± 0.05
R_Zr (Å)	3.48 ± 0.1
σ^2_O$_1$ (Å^2)	0.002 ± 0.001
σ^2_O$_2$ (Å^2)	0.001 ± 0.004
σ^2_Zr (Å^2)	0.01 ± 0.01

*Figure S14. Left) Pictorial representation of the DFT-optimized **Cu^{I}** structure, in which the SS contributions are highlighted with coloured circles. Right) results of the fits performed on the k^2-weighted FT-EXAFS experimental spectrum of MCR-derived **Cu^{I}** species.*

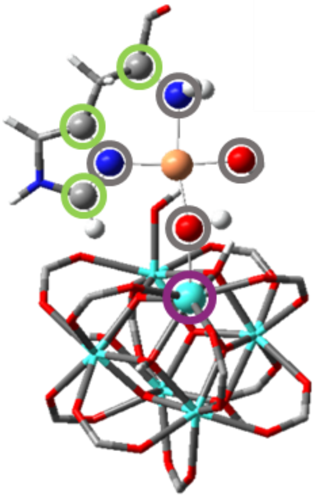

EXAFS parameters	Cu^{II}-his
$N°_{par}/N°_{ind}$	**9/17**
R_{factor}	0.03
S_0^2	1.0
ΔE (eV)	-7 ± 2
R_N,O (Å)	1.98 ± 0.01
R_C (Å)	2.74 ± 0.09
R_Zr (Å)	3.98 ± 0.12
σ^2_N,O (Å^2)	0.001 ± 0.001
σ^2_C (Å^2)	0.01 ± 0.01
σ^2_Zr (Å^2)	0.005 ± 0.002

*Figure S15. Left) Pictorial representation of the DFT-optimized **Cu^{II}-his** structure, in which the SS contributions are highlighted with coloured circles. Right) results of the fits performed on the k^2-weighted FT-EXAFS experimental spectrum of MCR-derived **Cu^{II}-his** species.*

According to the local environment in the **Cu^{II}-his** structure, we adopted a different parametrization with respect to the previous cases. The first shell involved one O atom of the defective node, one extra-ligand O atom, and two N atoms of the histidine unit, which are parametrized with the same ΔR and σ^2 and with average distance of Reff=1.98 Å, denoted as N,O. In the second coordination sphere, three C atoms belonging to histidine (C) at Reff=2.74 Å were considered, while the closest Zr atom giving a SS contribution has been included with specific ΔR_{Zr} and σ^2_{Zr} parameters, as reported in the previous cases.

7.3 *Additional results on EXAFS fitting for Cu^{I}-his*

We conducted an EXAFS fitting analysis using the DFT-optimized structure featuring Cu^{I} ion coordinated to both the histidine unit and the defective Zr site. The aim was to show that the MCR-derived pure **Cu^{I}** species can be described mainly with the defective Zr model **Cu^{I}** in Figure 4 obtained from the reduction

of **Cu^{II}-HC** and **Cu^{II}-LC** sites. The proposed DFT structure is reported in Figure S16, while the obtained EXAFS fitting results are presented in Figure S17.

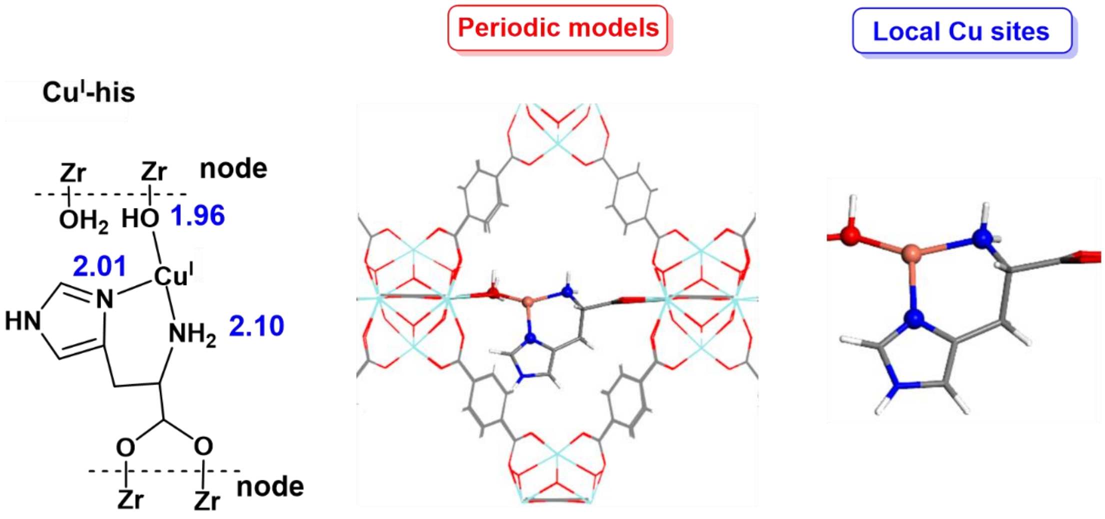

Figure S16. Cu^I-his DFT-optimized structure, involving Cu^I ions coordinated to the histidine ligand in the UiO-66-Cu MOFs. Bond distances in Å from the Cu centre are reported in blue.

As expected, certain fitting parameters (highlighted in yellow in the Table reported in the right part of Figure are unphysical: the Debye-Waller factors (σ^2) of C and Zr present excessively high values, and the variation of interatomic distances (ΔR) of the C atoms are higher than the values reported in literature (should remain of the order of maximum 0.15 Å),[6] which would result in unrealistic deformations of the DFT-optimized staring model. Consequently, we can conclude that this analysis is unsuccessful, thereby ruling out the presence of these species in the mixture to a significant extent.

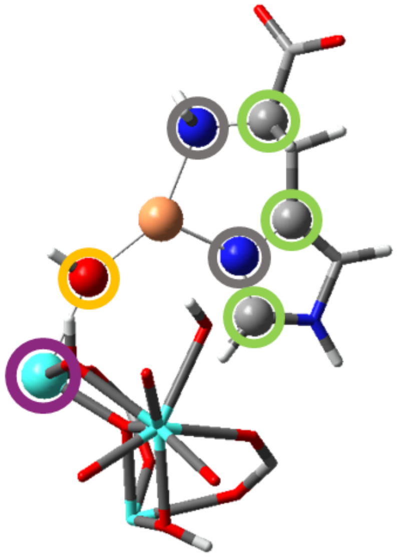

EXAFS parameters	Cu^I-his
$N°_{par}/N°_{ind}$	**7/15**
R_{factor}	0.05
S_0^2	1.0
ΔE (eV)	4 ± 2
ΔR_O (Å)	-0.11 ± 0.04
ΔR_N (Å)	-0.06 ± 0.03
ΔR_C (Å)	0.4 ± 0.7
ΔR_Zr (Å)	0.14 ± 0.19
σ^2_O (Å^2)	0.002 ± 0.004
σ^2_N (Å^2)	0.002 ± 0.004
σ^2_C (Å^2)	0.08 ± 0.16
σ^2_Zr (Å^2)	0.02 ± 0.02

Figure S17. Left) Pictorial representation of the DFT-optimized Cu^I-his structure, in which the SS contributions are highlighted with coloured circles. Right) results of the fits performed on the k^2-weighted FT-EXAFS experimental spectrum of MCR-derived Cu^I species.

7.4 Comparison of geometries with periodic and cluster models

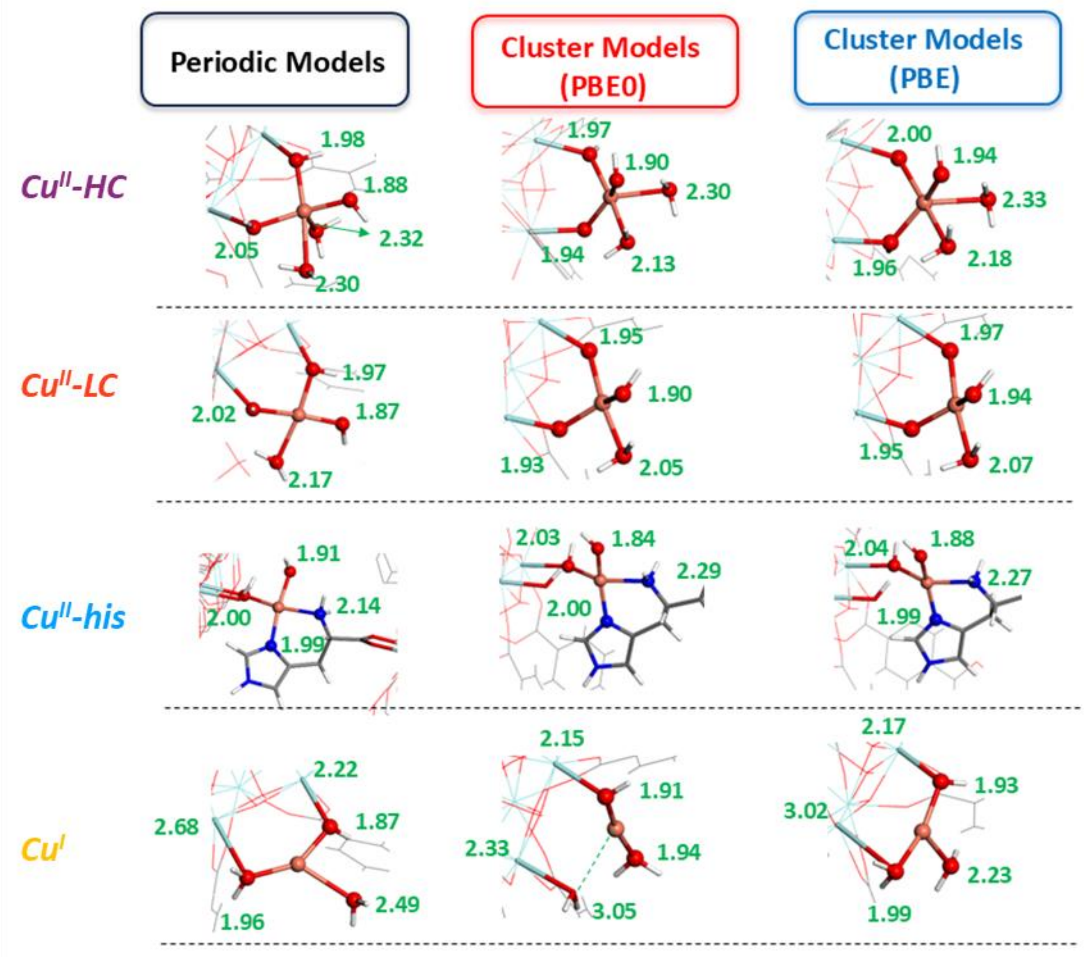

Figure S18. Bond distances in periodic and cluster models optimized using the PBE0 and PBE functionals, with bond lengths (in Å) displayed in green.

As depicted in Figure S18, the Cu^{II} cluster models generally replicate the periodic models well using either the PBE or PBE0 functional. However, the cluster models tend to underestimate the Cu–OH_2 bond lengths and overestimate the Cu–N_{amine} bond lengths. Additionally, both cluster models fail to capture the geometries observed in periodic models. For instance, the cluster model optimized with the PBE0 functional exhibits a linear bidentate configuration, whereas the periodic model shows a tridentate configuration. Additionally, in the PBE-optimized cluster, the water molecule has a 3.02 Å interatomic distance from the Zr atom, indicating de-coordination, which is not observed in periodic models.

8 References

1. Aunan, E.; Finelli, V.; Prodinger, S.; Cao, N.; Garetto, B.; Deplano, G.; Njoroge, P. N.; Signorile, M.; Borfecchia, E.; Lillerud, K. P.; Nova, A.; Bordiga, S.; Olsbye, U. Partial oxidation of cyclohexene over histidine-modified Cu-UiO-66 under aerobic conditions. *J. Catal*. **2024**, *438*, 115722.
2. Ternero-Hidalgo, J. J.; Guerrero-Pérez, M. O.; Rodríguez-Mirasol, J.; Cordero, T.; Bañares, M. A.; Portela, R.; Bazin, P.; Clet, G.; Daturi, M. Operando Reactor-Cell with Simultaneous Transmission FTIR and Raman Characterization (IRRaman) for the Study of Gas-Phase Reactions with Solid Catalysts. *Anal Chem* **2020**, *92 (7)*, 5100.

3. Zabinsky, S. I.; Rehr, J. J.; Ankudinov, A.; Albers, R. C.; Eller, M. J. Multiple-scattering calculations of x-ray-absorption spectra. *Phys. Rev. B*. **1995**, *52*, 2995-3009.
4. Ravel, B.; M. Newville, M. ATHENA, ARTEMIS, HEPHAESTUS: data analysis for X-ray absorption spectroscopy using IFEFFIT. *J. Synch. Radiat.* **2005**, *12*, 537-541.
5. Haddad, L.; Gianolio, D.; Dunstan, D. J.; Sapelkina, A. Quantifying Intuition: Bayesian Approach to Figures of Merit in EXAFS Analysis of Magic Size Clusters. *Nanoscale* **2024**, *16*, 5768-5775.
6. Molokova, A. Y.; Salusso, D.; Borfecchia, E.; Wen, F.; Magliocco, S.; Bordiga, S.; Janssens, T. V. W.; Lomachenko, K. A.; Berlier, G. The chemical nature of SO2 poisoning of Cu-CHA-based SCR catalysts for NOx removal in diesel exhausts. *Catal. Sci. Technol.* **2024**, *14*, 5989-5995.
